# Supplementary material for: Circular olefin copolymers made de novo from ethylene and α-olefins
Source: Nat Commun. 2024 Feb 17;15:1462. doi: 10.1038/s41467-024-45219-w (PMC10874424; doi:10.1038/s41467-024-45219-w)
Supplement: Supplementary file 1 — Supplementary Information [file 41467_2024_45219_MOESM1_ESM.pdf]

Supplementary Information

Circular olefin copolymers made *de novo* from ethylene and α-olefins

Xing-Wang Han, Xun Zhang, Youyun Zhou, Aizezi Maimaitiming, Xiu-Li Sun, Yanshan Gao,*
Peizhi Li, Boyu Zhu, Eugene Y.-X. Chen,* Xiaokang Kuang and Yong Tang*

Correspondence to: Yong Tang, tangy@sioc.ac.cn; Yanshan Gao, gaoyanshan@sioc.ac.cn; Eugene Y.-X. Chen, eugene.chen@colostate.edu

Table of Contents

S1. Supplementary Methods……S2

Synthesis and characterization of ((6-bromohexyl)oxy)triisopropylsilane……S2

Synthesis and characterization of dialkylzinc Zn[$(CH_2)_6$OTIPS]$_2$……S2

NMR spectra of the telechelic macromonomers before and after deprotection ……S3

NMR spectra of the recyclable polymers, *r*POs and *r*OBCs……S9

NMR spectra of the samples in closed-loop recycling experiments……S14

GPC characterizations of *t*POs, *r*POs, *r*OBCs ……S17

MALDI-TOF characterizations of *t*POs……S28

TGA measurements of *r*PO$_0$-1, *r*PO$_{3.1}$, *r*PO$_{9.6}$, *r*PO$_{8.9}$, *r*OBC$_{7.7}$ and *c*PO$_{11.2}$……S30

DSC characterizations of *r*POs and *r*OBCs……S34

Powder X-ray characterizations of *r*POs and *r*OBCs……S40

S2. Supplementary Tables ……S43

Dogbone tensile specimens and tensile test ……S43

Supplementary Table 1 Data of tensile stress-strain testing……S44

Supplementary Table 2 Data of Young's modulus of selected samples……S44

Supplementary Table 3 Data of lap shear analysis……S44

References ……S45

S1. Supplementary Methods

Synthesis and characterization of [(6-bromohexyl)oxy]triisopropylsilane

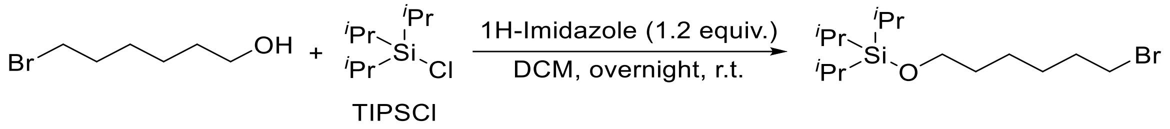

This compound was prepared according to literature procedures.[1] 6-Bromohexan-1-ol (200 mmol, 36.214 g) was added to a solution of TIPSCl (200 mmol, 7.712 g) and imidazole (240 mmol, 16.339 g, 1.2 equiv.) in dry CH_2Cl_2 to start the reaction at room temperature. After overnight (~ 12 h) reaction, the mixture was diluted with Et_2O, washed with water, saturated $NaHCO_3$, and then brine, dried over Na_2SO_4, and concentrated on a rotary evaporator. The resulting crude product was purified by flash column chromatography with petroleum ether to give colorless liquid, 60.732 g, 90% isolated yield. ^{1}H NMR (600 MHz, $CDCl_3$) δ 3.68 (t, J = 6.4 Hz, 2H), 3.41 (t, J = 6.8 Hz, 2H), 1.87 (dt, J = 7.0 Hz, 2H), 1.61 – 1.51 (m, 2H), 1.48 – 1.37 (m, 4H), 1.08 – 1.05 (m, 21H).

Synthesis and characterization of dialkylzinc Zn[$(CH_2)_6$OTIPS]$_2$

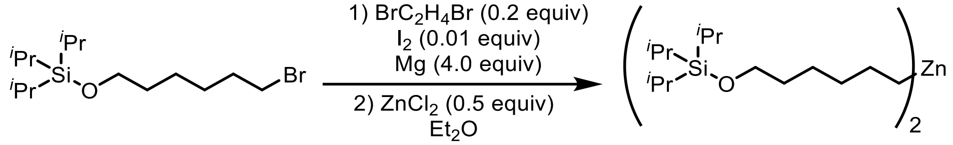

This dialkylzinc *f*CTA was prepared following literature procedures.[2] A flask was flame-dried and then cooled under vacuum. The flask was purged with N_2 and charged with Mg turnings (3.888 g, 160.0 mmol, 4.0 equiv). Anhydrous Et_2O (5 mL) was added to fully cover the Mg turnings. The mixture was stirred vigorously, and 1,2-dibromoethane (1.502 g, 8.0 mmol, 0.2 equiv.) was added dropwise as the mixture started bubbling due to the reaction exotherm. The flask was allowed to cool to room temperature, and I_2 (ca. 0.102 g, 0.4 mmol, 0.01 equiv) was added. A solution of ((6-bromohexyl)oxy)triisopropylsilane (13.496 g, 40.0 mmol, 1.0 equiv.) in anhydrous Et_2O (60 mL, ca. 0.67 M) was prepared, and a small portion (ca. 1 mL) of this solution was added to the reaction mixture. The mixture was then gently heated until the dark brown color faded (consumption of I_2). At this time, the remaining portion of the solution was added dropwise. Upon completion of the addition, the mixture was stirred for an additional hour. The resulting Grignard reagent was titrated with a solution of I_2 in Et_2O three times to obtain the accurate concentration of RMgBr in Et_2O (43.4 mL, 34.026 mmol, 0.784 M). In a separate round-bottom flask, $ZnCl_2$ (2.319 g, 17.0 mmol,

0.5 equiv.) was flame-dried under vacuum. Upon cooling, the flask was purged with N_2, and 3.0 mL anhydrous Et_2O was added. The mixture was stirred and the prepared Grignard reagent in Et_2O was added dropwise to the solution of $ZnCl_2$. After stirring at room temperature for 1 hour, Et_2O was removed under reduced pressure and the resulting $Zn[(CH_2)_6OTIPS]_2$ was extracted with *n*-hexane. The obtained alkylzinc compound (9.374 g, yield = 95%) was used as a stock solution (0.5 M in *n*-hexane) for the next step olefin polymerizations without further purification.

NMR spectra of the telechelic macromonomers before and after deprotection

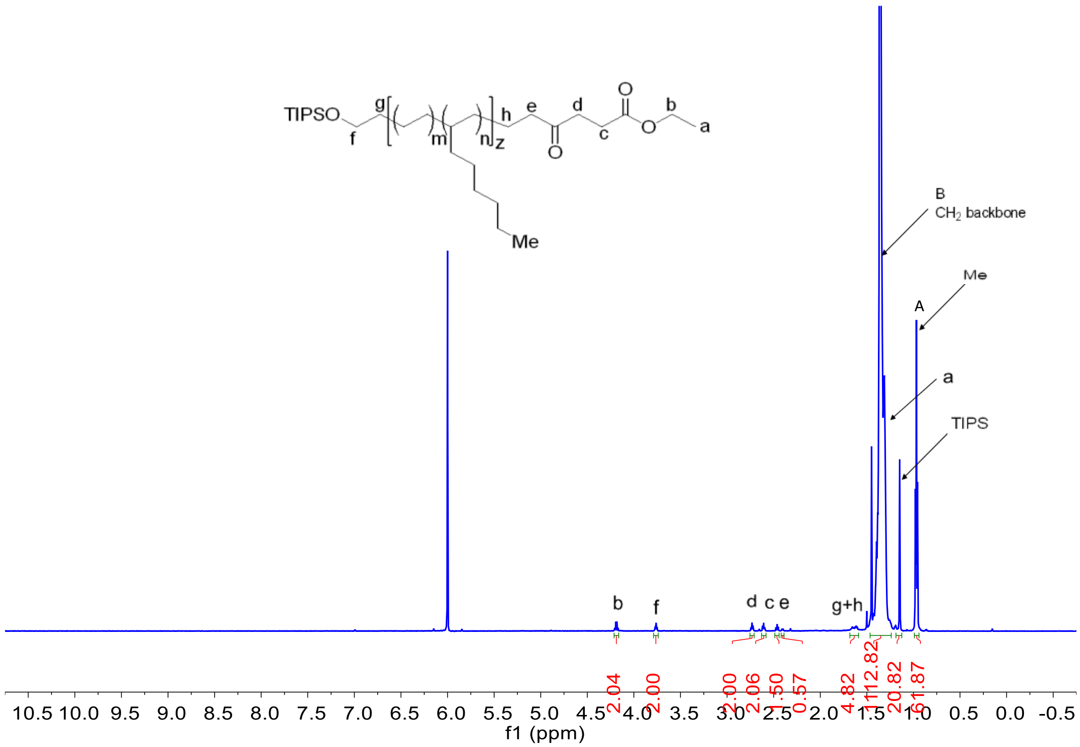

Supplementary Figure 1. ^{1}H NMR of tPO$_{8.9}$ before deprotection in TCE-d_2 (110 °C) , Table 1 entry 5.

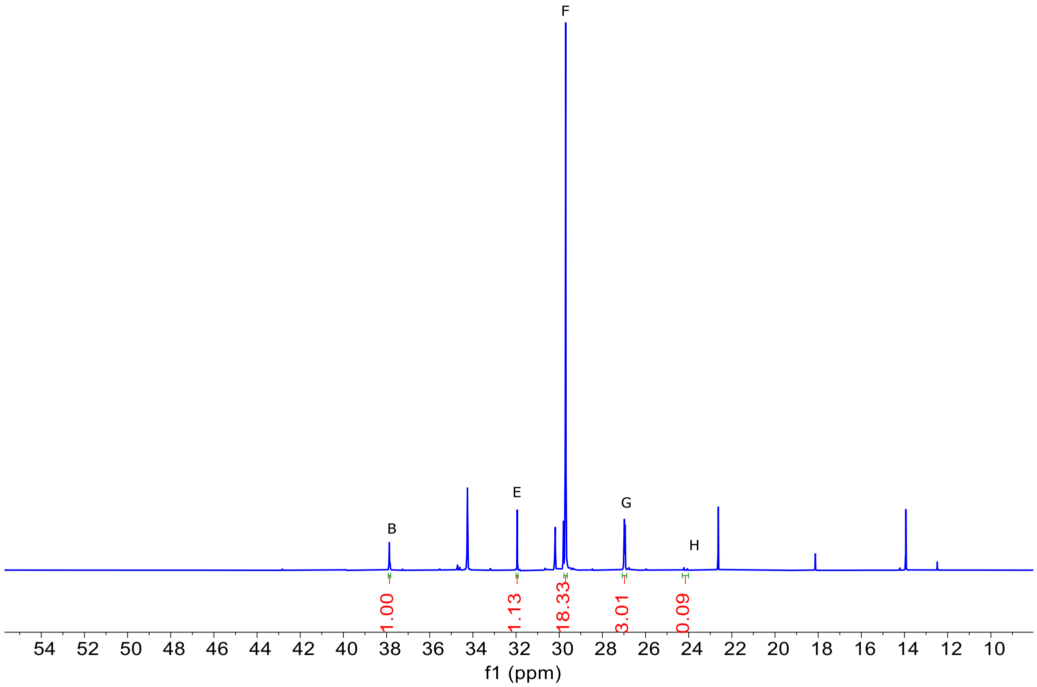

Supplementary Figure 2. ^{13}C NMR of *t*$PO_{8.9}$ before deprotection in TCE-d_2 (110 °C) , Table 1 entry 5.

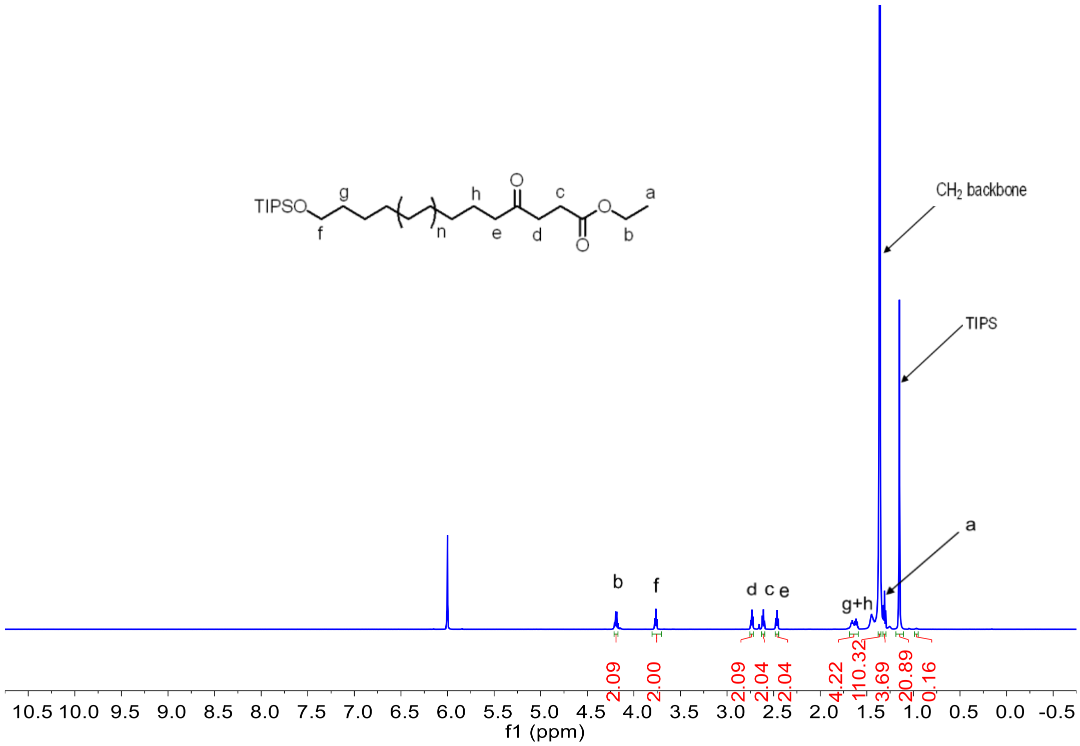

Supplementary Figure 3. ^{1}H NMR of *t*PO_0-1 before deprotection in TCE-d_2 (110 °C), Table 1 entry 1.

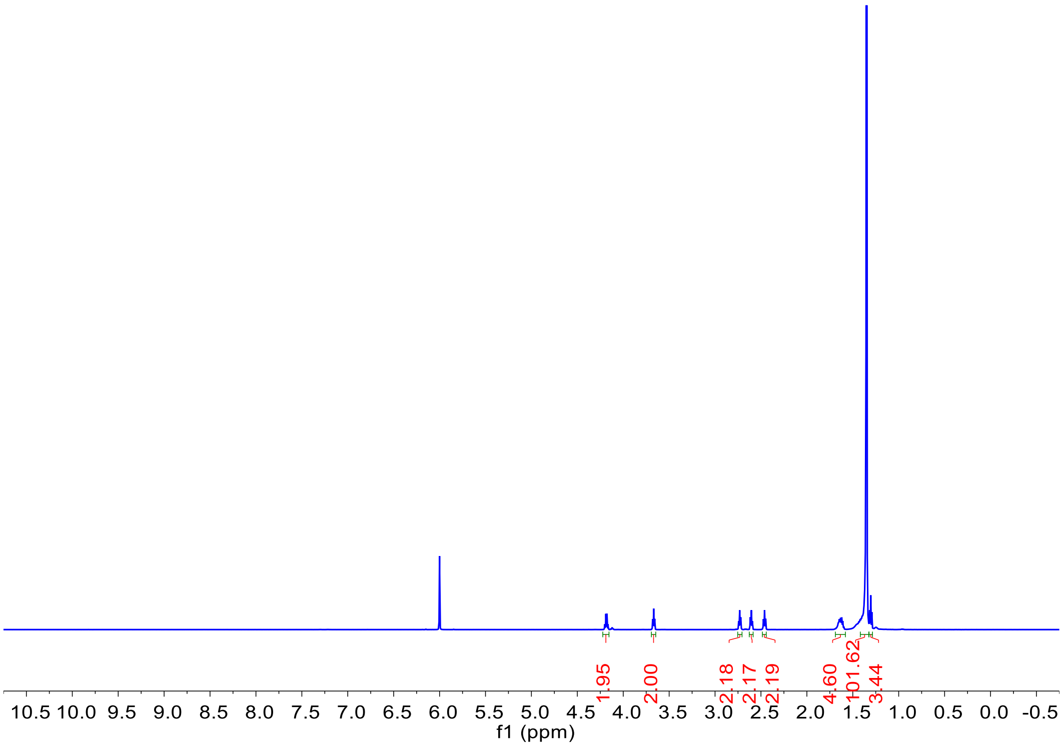

Supplementary Figure 4. ^{1}H NMR of *t*PO_0-1 after deprotection in TCE-d_2 (110 °C), Table 1 entry 1.

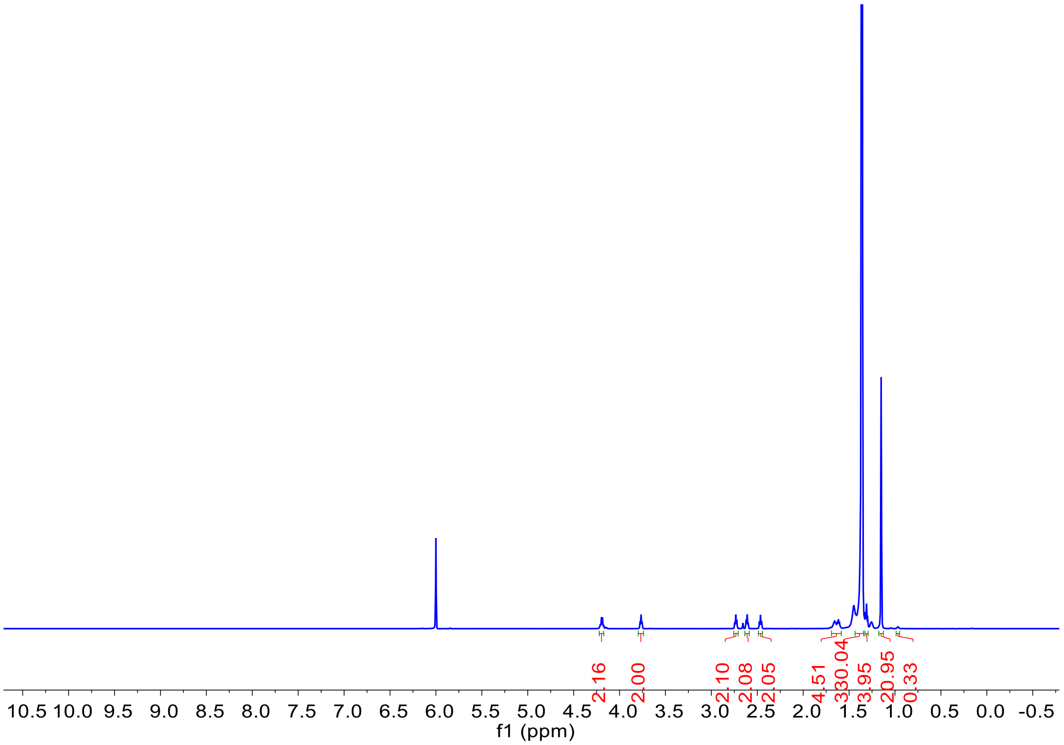

Supplementary Figure 5. ^{1}H NMR of *t*PO$_0$-2 before deprotection in TCE-d_2 (110 °C), Table 1 entry 2.

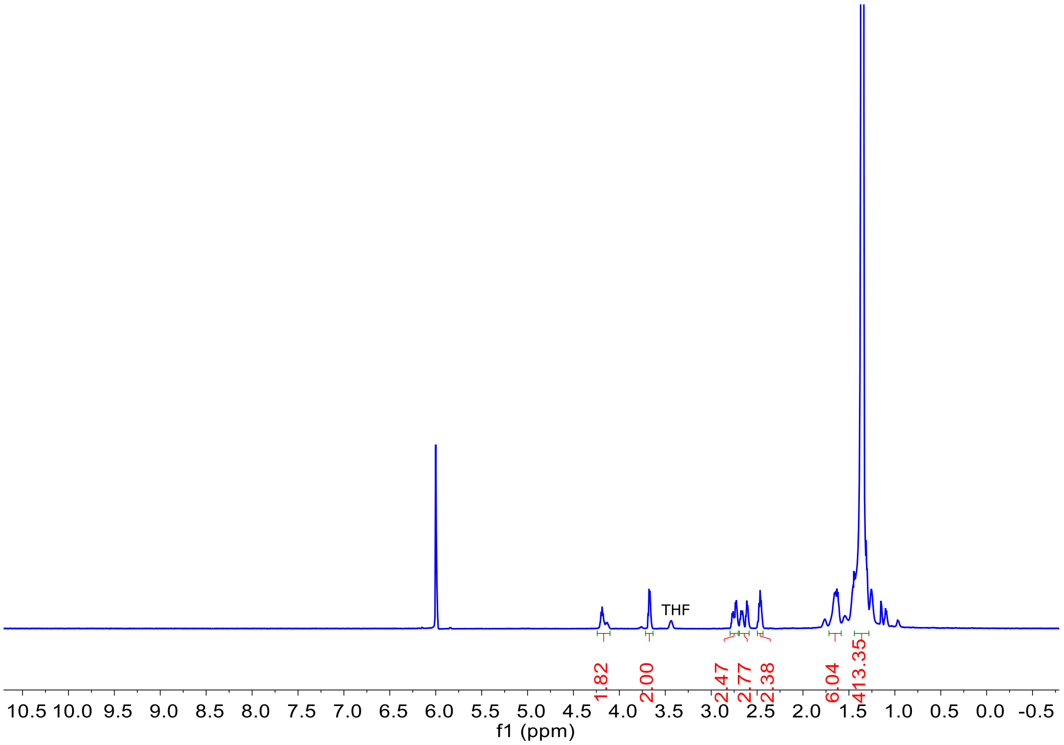

Supplementary Figure 6. ^{1}H NMR of *t*PO$_0$-2 after deprotection in TCE-d_2 (110 °C) , Table 1 entry 2.

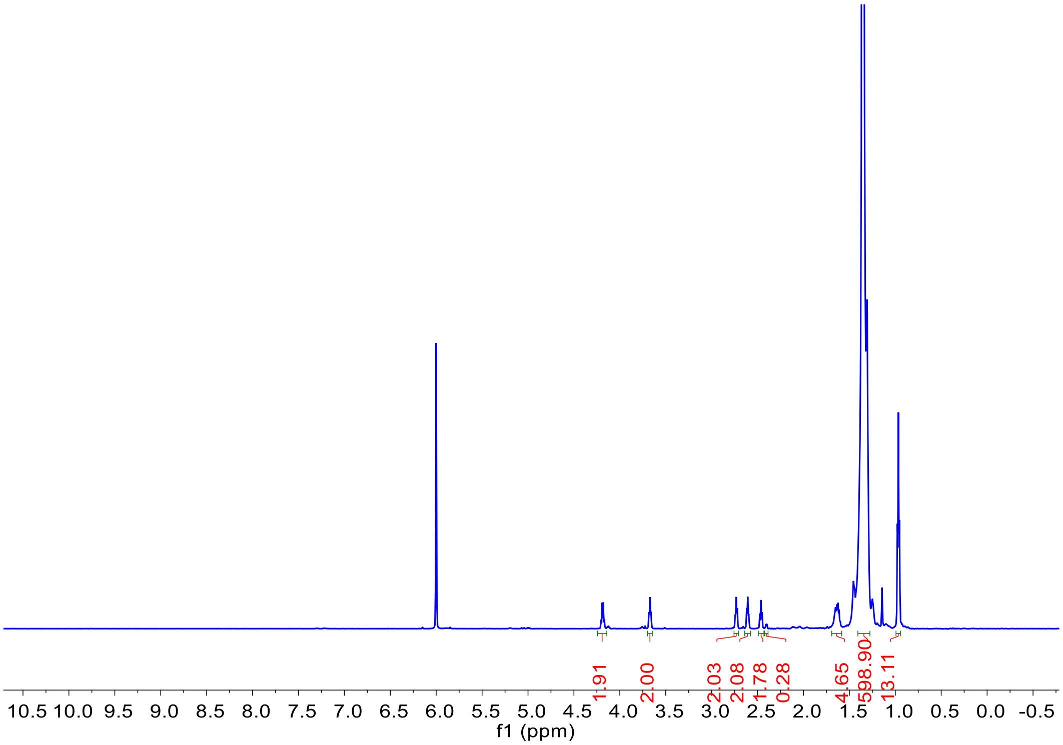

Supplementary Figure 7. ^{1}H NMR of *t*$PO_{3.1}$ after deprotection in TCE-d_2 (110 °C), Table 1 entry 3.

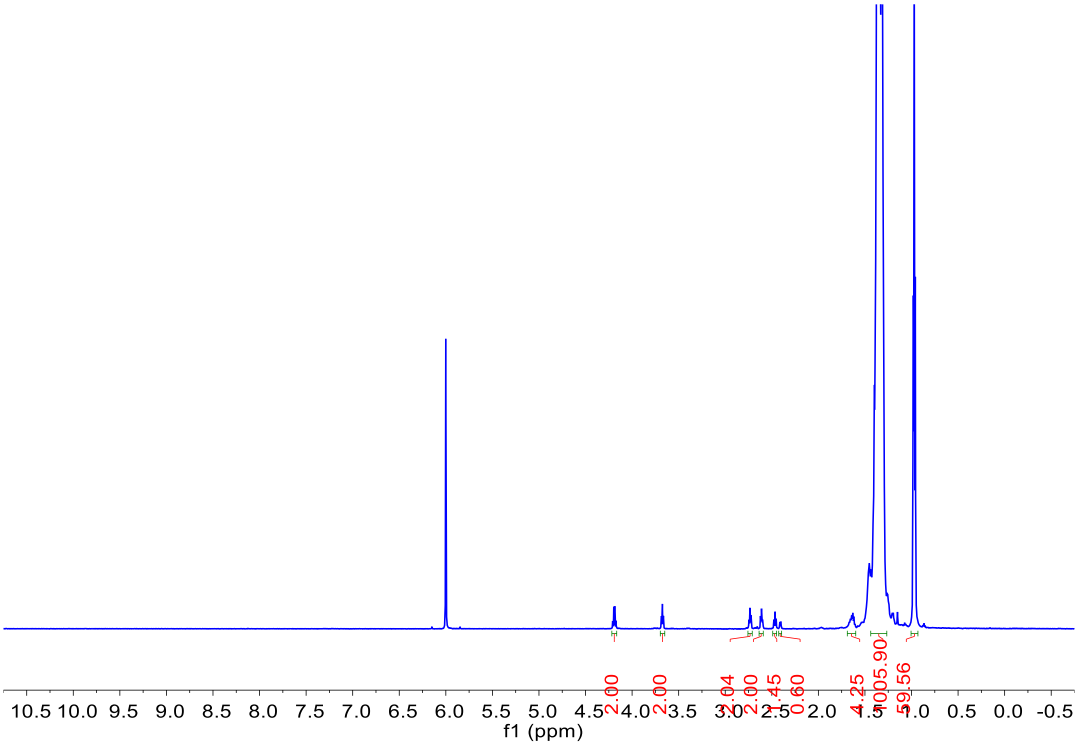

Supplementary Figure 8. ^{1}H NMR of $t\mathrm{PO}_{9.6}$ after deprotection in TCE-d_2 (110 °C), Table 1 entry 4.

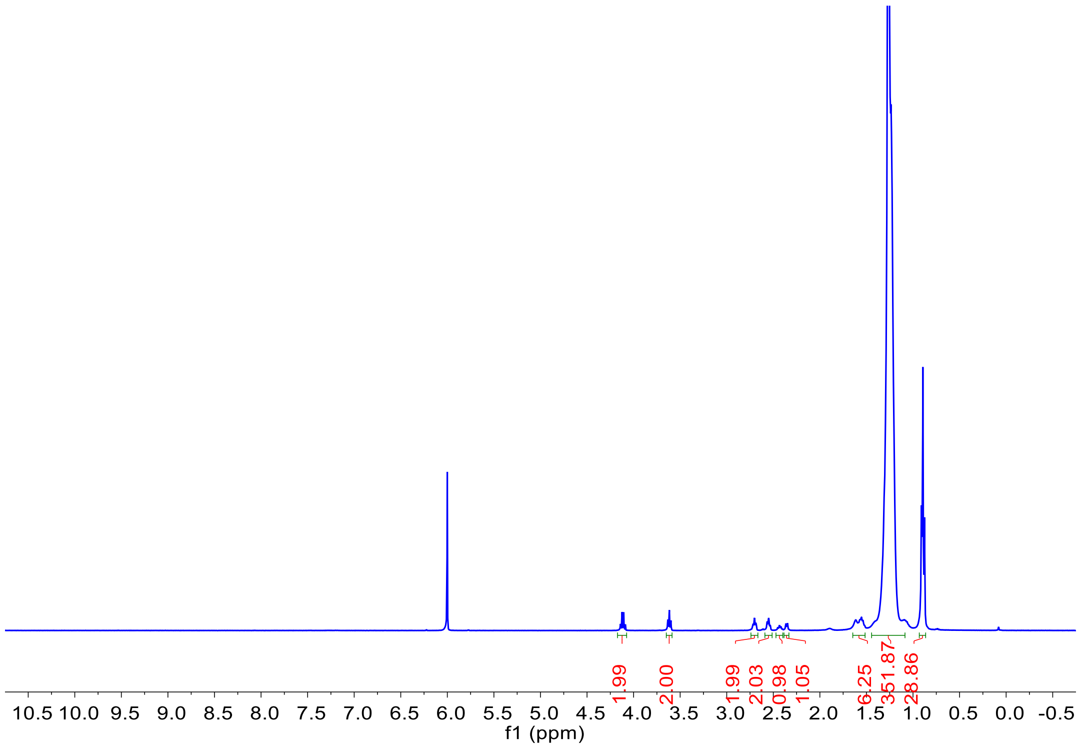

Supplementary Figure 9. ^{1}H NMR of $t\mathrm{PO}_{14.8}$ after deprotection in TCE-d_2 (110 °C) , Table 1 entry 6.

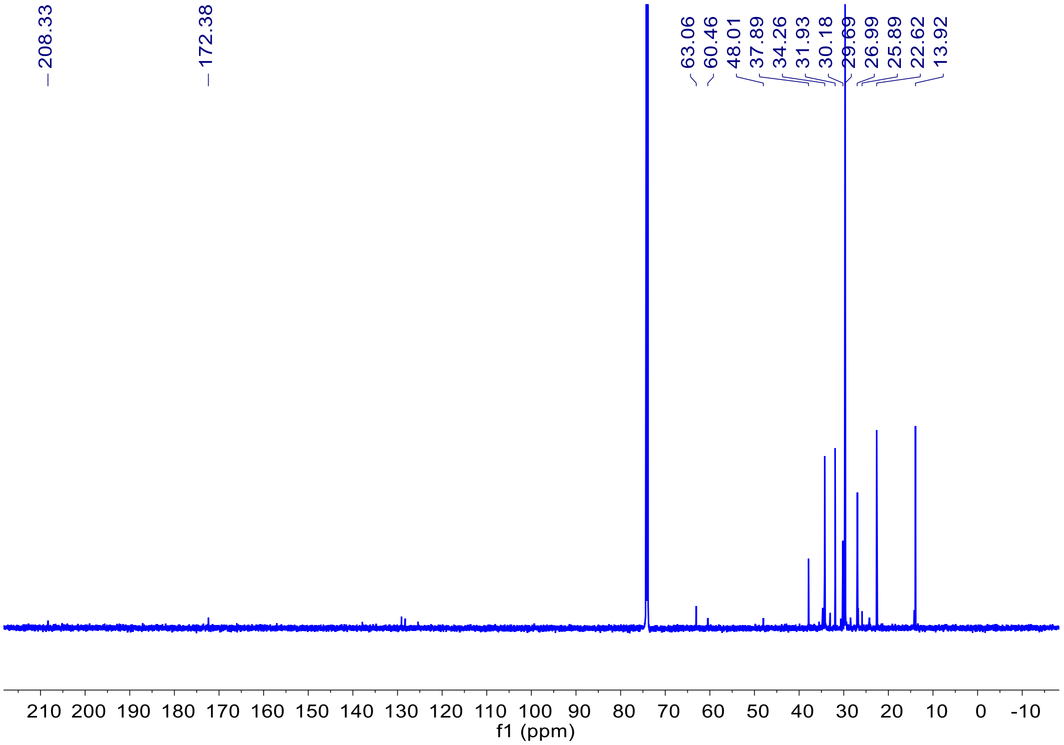

Supplementary Figure 10. ^{13}C NMR of *t*$PO_{14.8}$ after deprotection in TCE-d_2 (110 ^{o}C) , Table 1 entry 6.

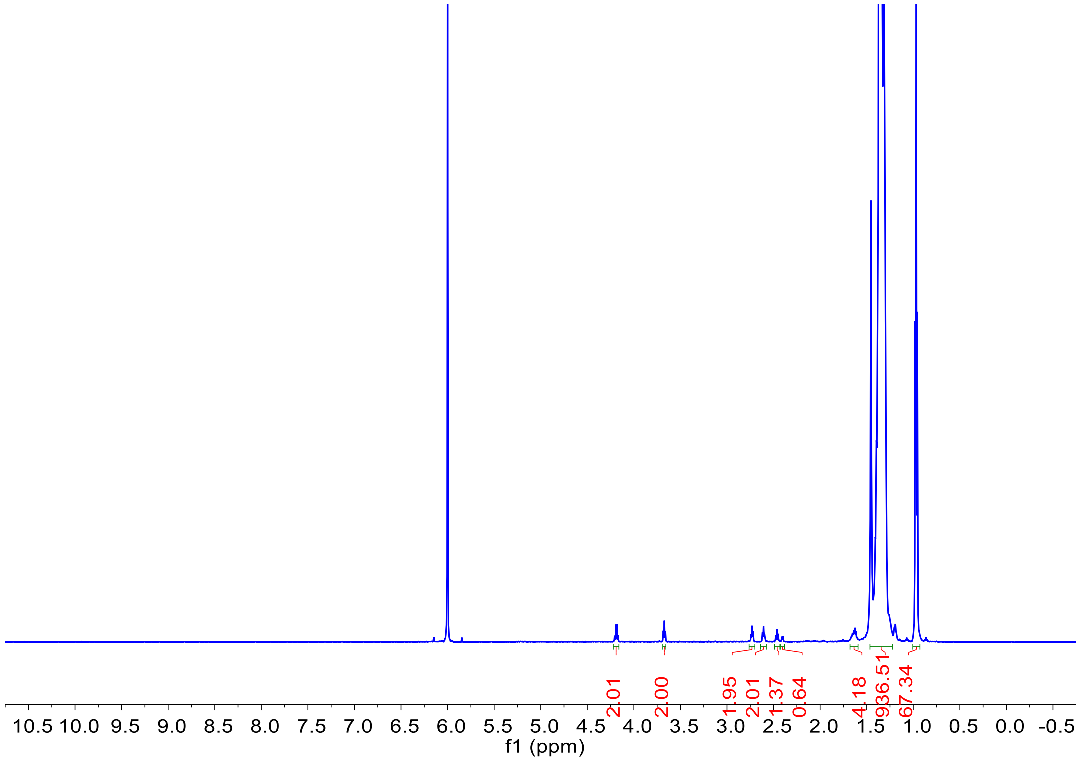

Supplementary Figure 11. ^{1}H NMR of *t*$PO_{12.2}$ after deprotection in TCE-d_2 (110 °C) , Table 1 entry 7.

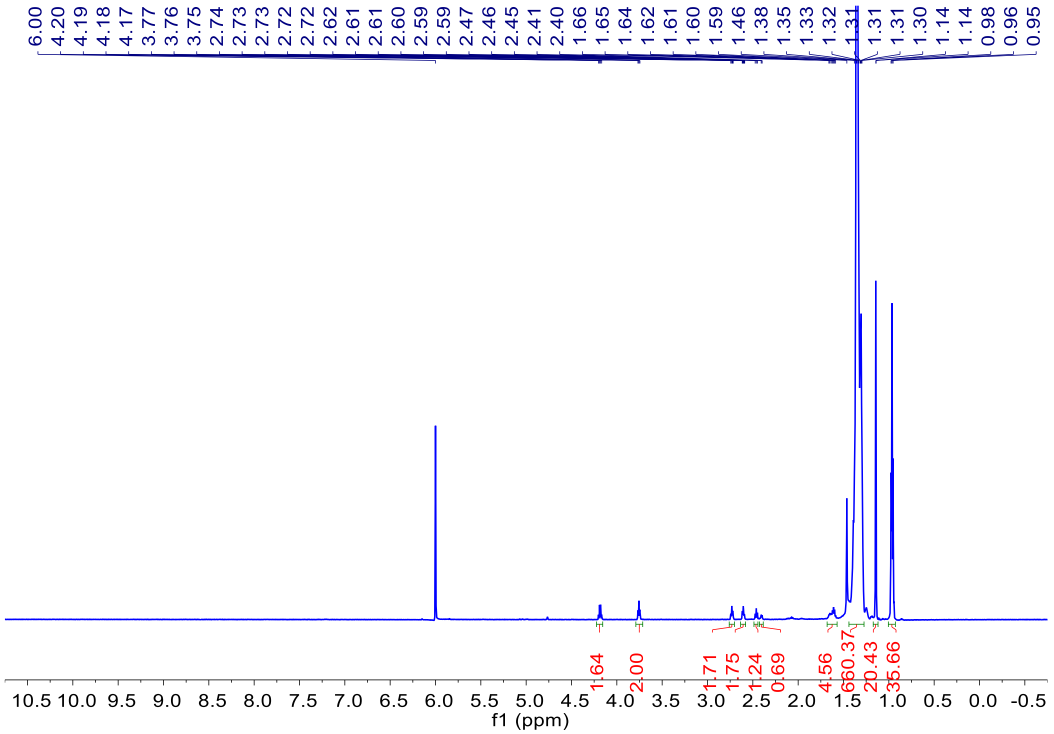

Supplementary Figure 12. ^{1}H NMR of *t*$PO_{12.2}$ before deprotection (without purification) in TCE-d_2 (110 °C).

NMR spectra of the recyclable polymers, *r*POs and *r*OBCs

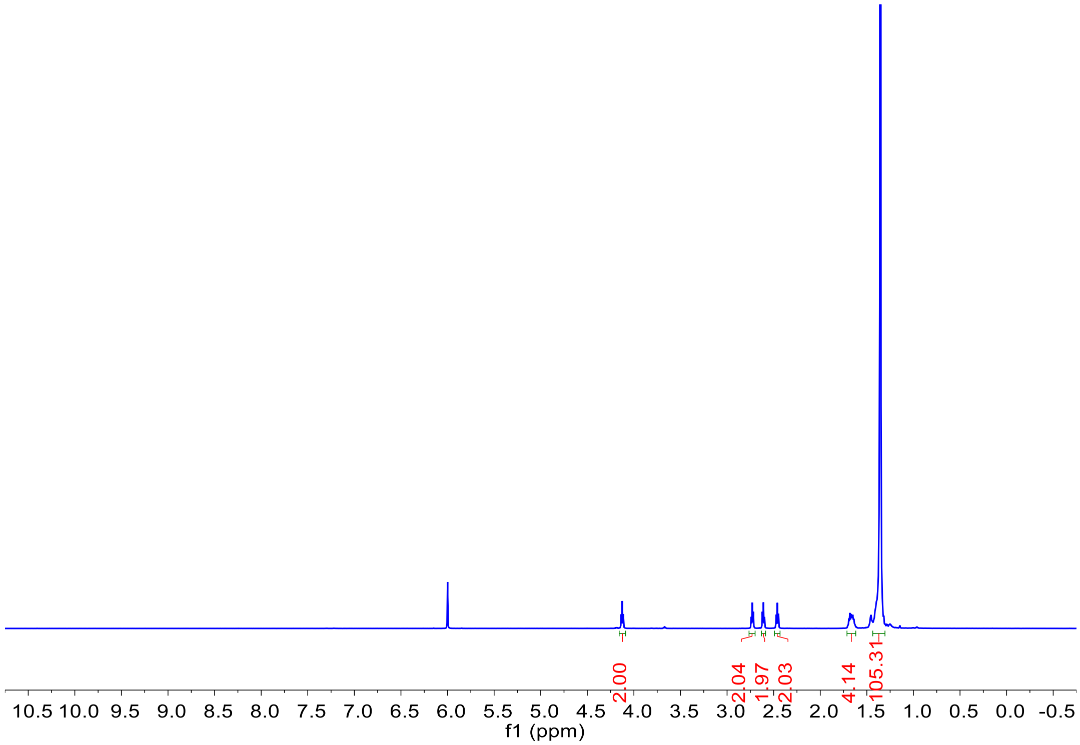

Supplementary Figure 13. ^{1}H NMR of *r*PO_0-1 in TCE-d_2 (110 °C) , Table 2 entry 1.

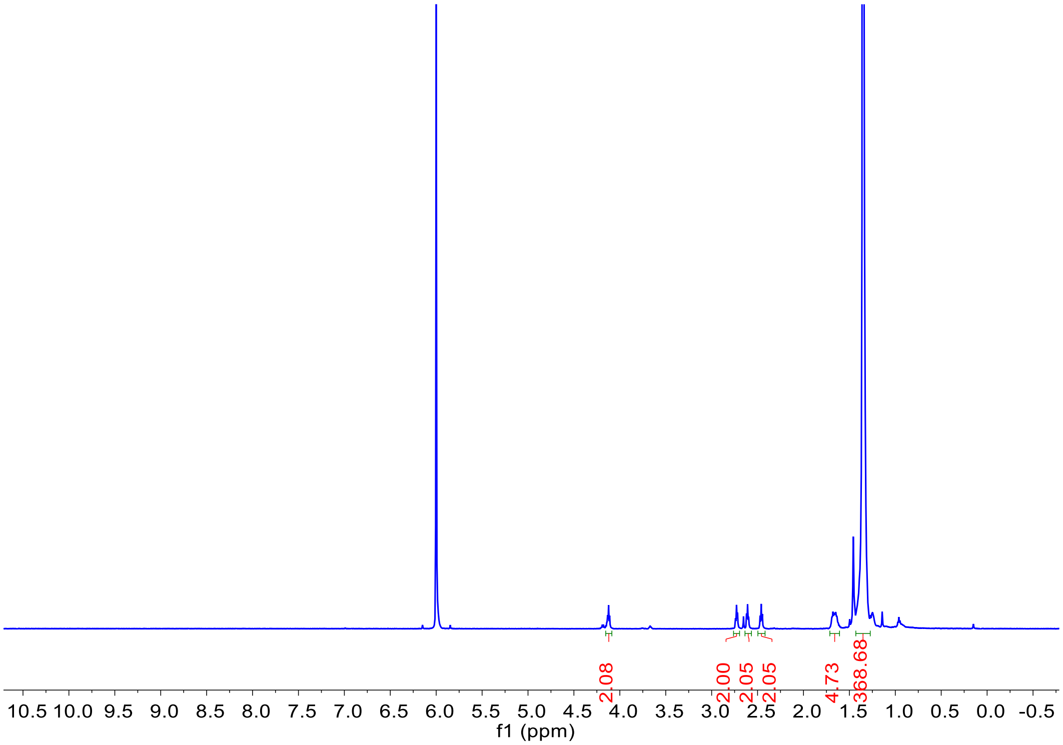

Supplementary Figure 14. ^{1}H NMR of *r*PO_0-2 in TCE-d_2 (110 °C), Table 2 entry 2.

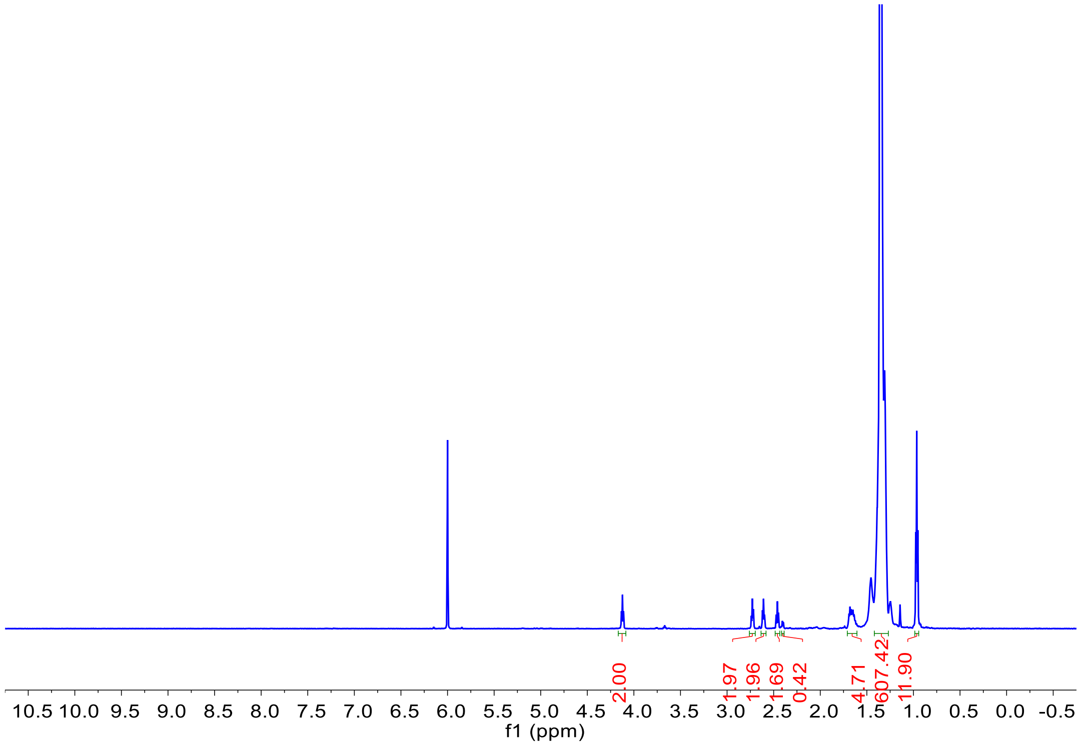

Supplementary Figure 15. ^{1}H NMR of rPO$_{3.1}$ in TCE-d_2 (110 °C) , Table 2 entry 3.

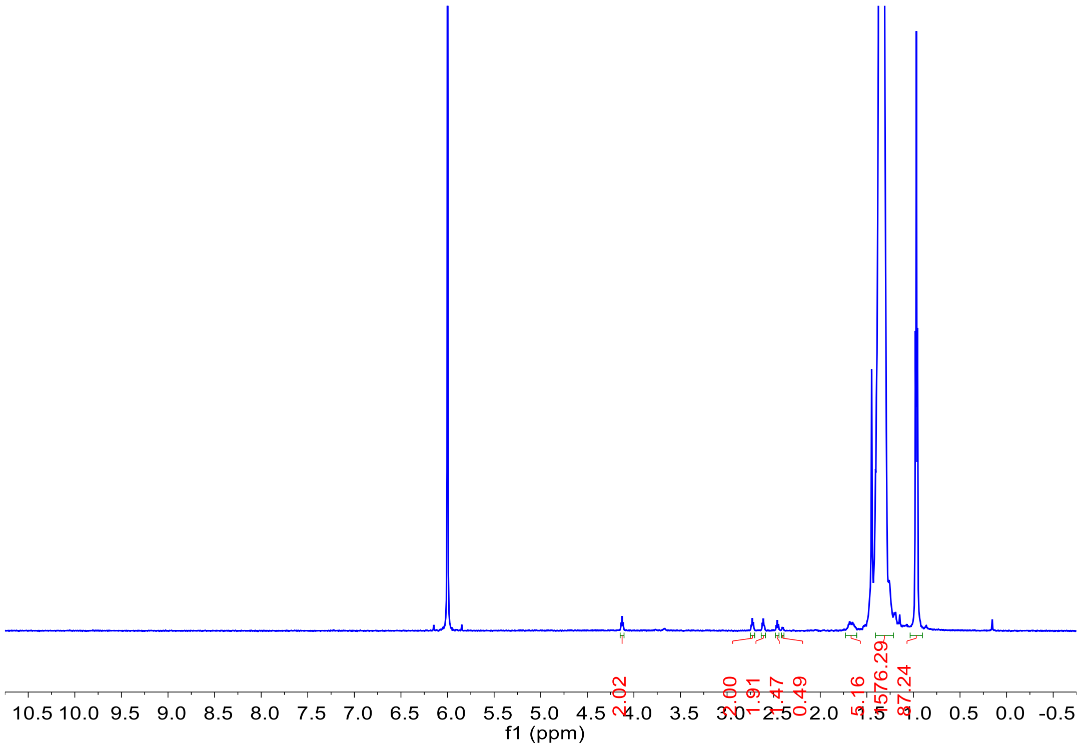

Supplementary Figure 16. ^{1}H NMR of rPO$_{8.9}$ in TCE-d_2 (110 °C), Table 2 entry 4.

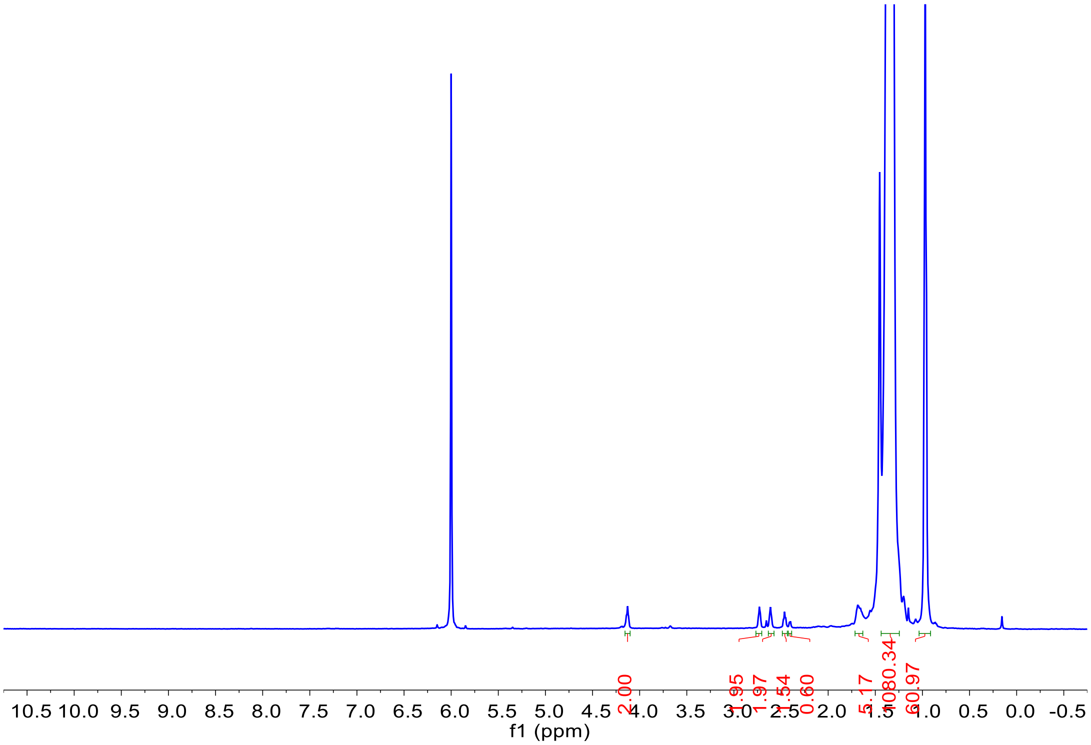

Supplementary Figure 17. ^{1}H NMR of rPO$_{9.6}$ in TCE-d_2 (110 °C) , Table 2 entry 5.

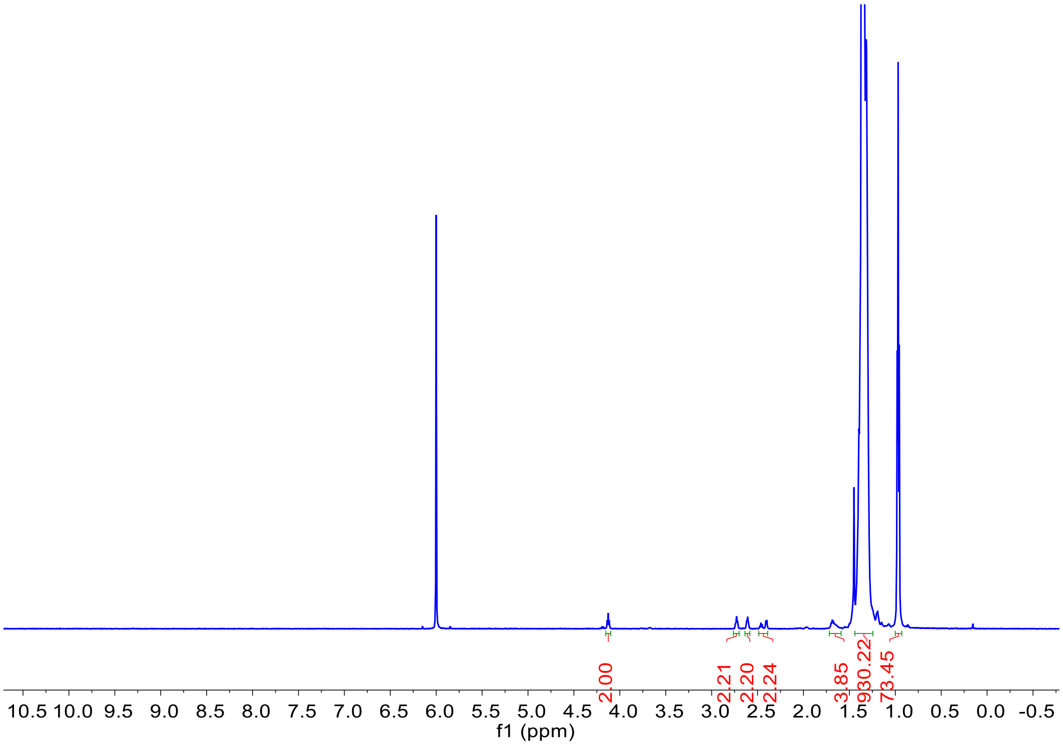

Supplementary Figure 18. ^{1}H NMR of rPO$_{12.2}$ in TCE-d_2 (110 °C), Table 2 entry 6.

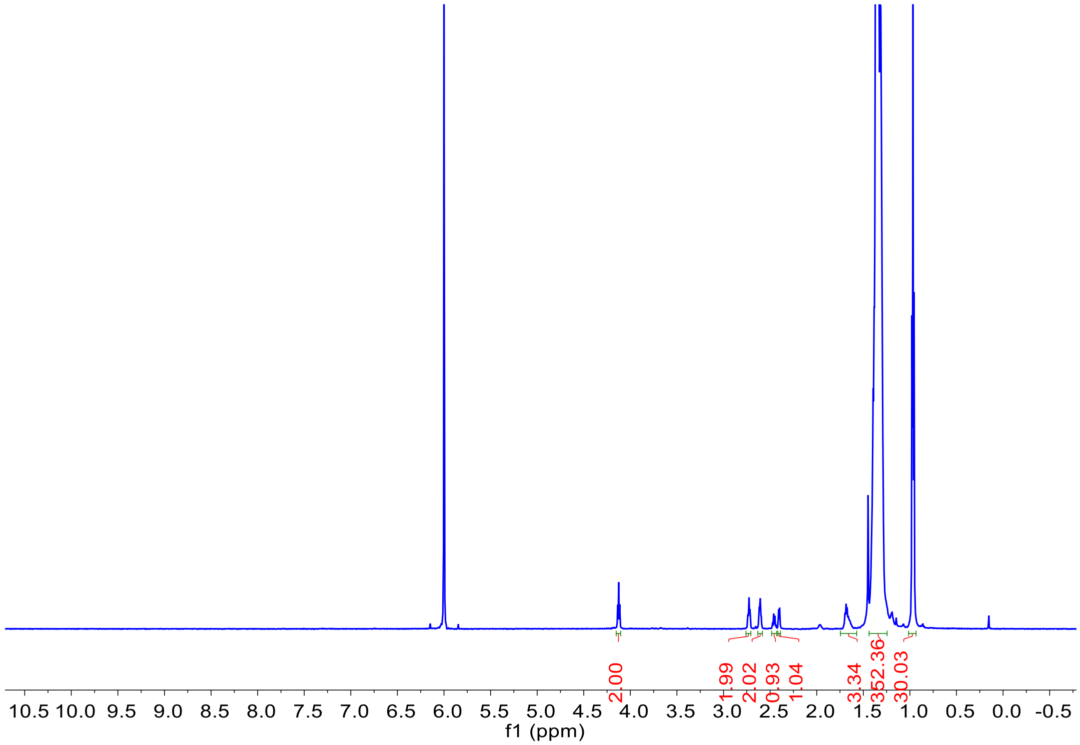

Supplementary Figure 19. ^{1}H NMR of *r*$PO_{14.8}$ in TCE-d_2 (110 °C), Table 2 entry 7.

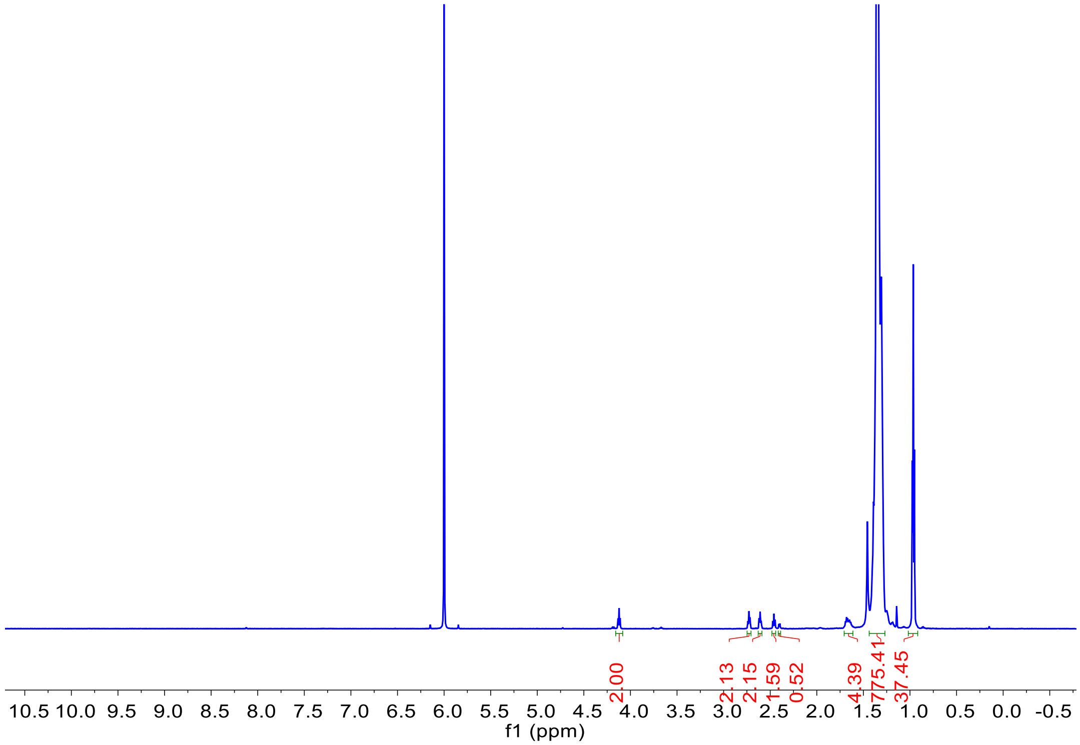

Supplementary Figure 20. ^{1}H NMR of *r*$OBC_{7.7}$ in TCE-d_2 (110 °C), Table 2 entry 8.

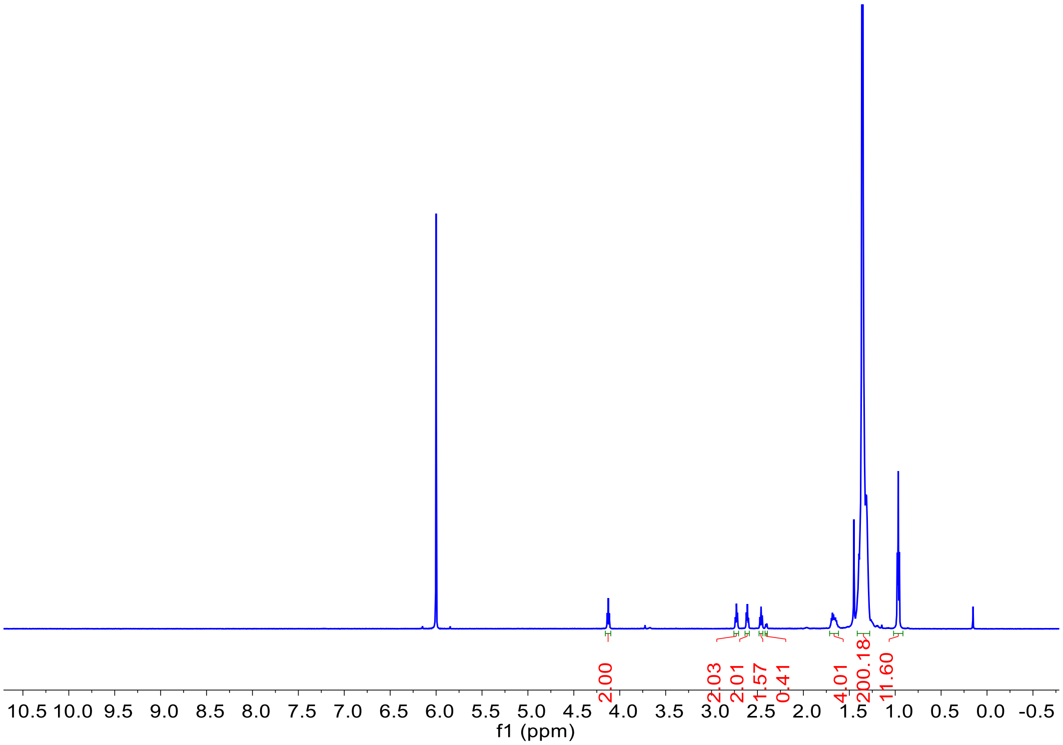

Supplementary Figure 21. ^{1}H NMR of *r*OBC$_{9.4}$ in TCE-d_2 (110 °C), Table 2 entry 9.

NMR spectra of the samples in closed-loop recycling experiments

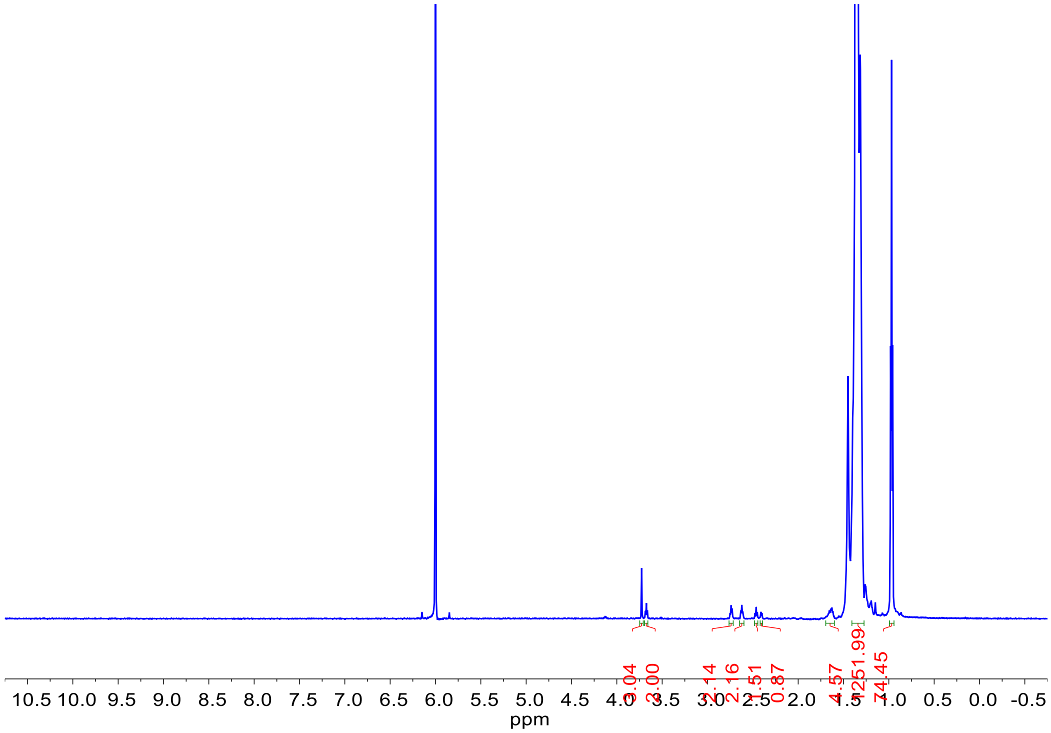

Supplementary Figure 22. ^{1}H NMR of *t*$PO_{9.6}$-cycle 1 in TCE-d_2 (110 ^{o}C), Figure 3A.

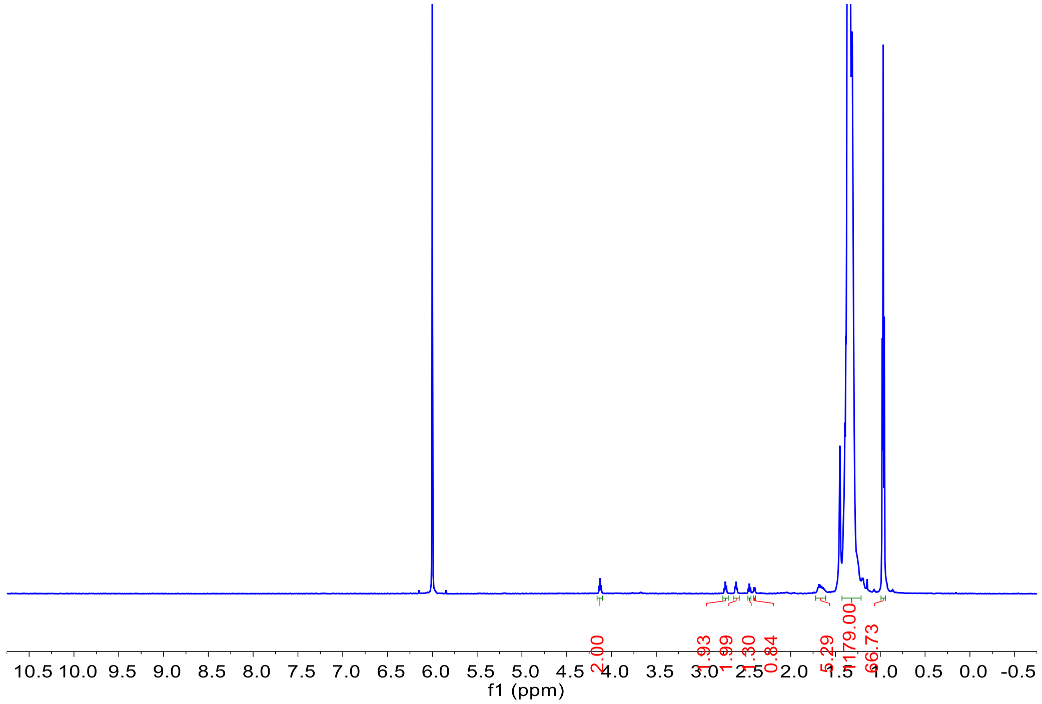

Supplementary Figure 23. ^{1}H NMR of *r*$PO_{9.6}$-cycle 1 in TCE-d_2 (110 ^{o}C), Figure 3A.

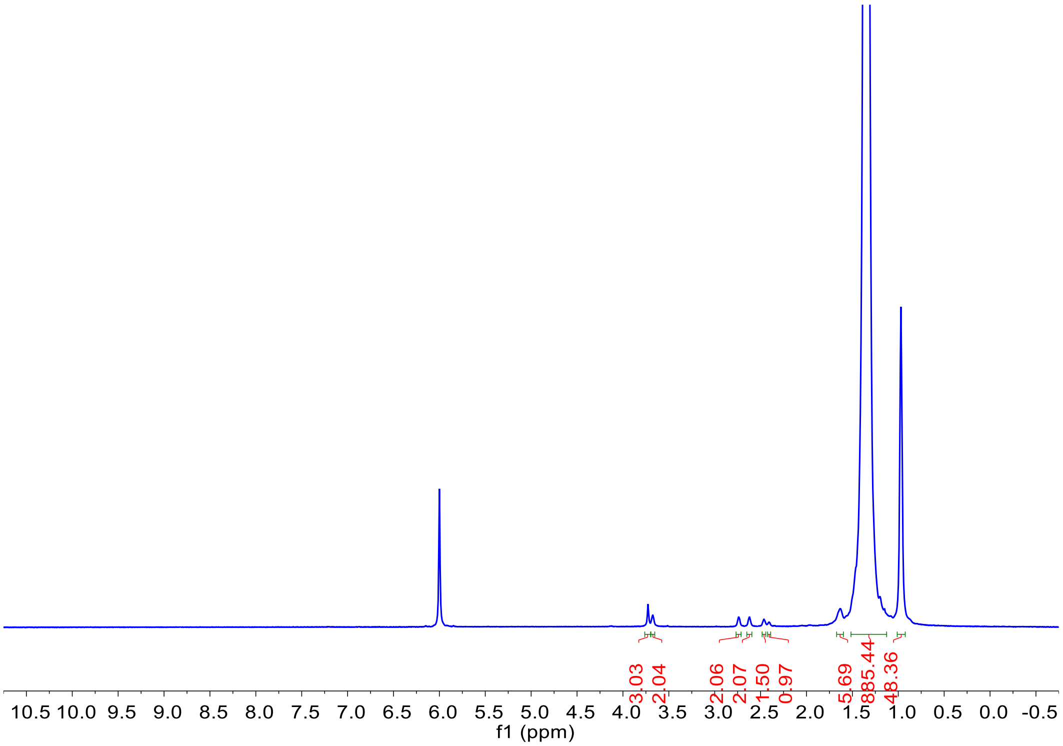

Supplementary Figure 24. ^{1}H NMR of *t*$PO_{9.6}$-cycle 2 in TCE-d_2 (110 ^{o}C), Figure 3A.

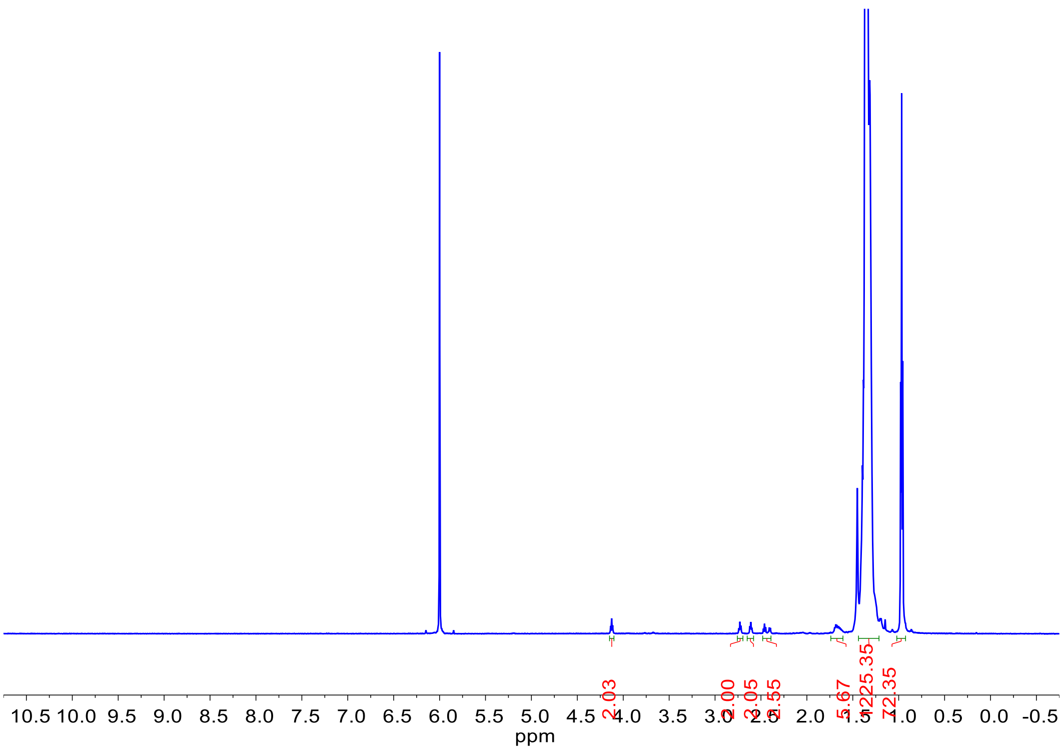

Supplementary Figure 25. ^{1}H NMR of *r*$PO_{9.6}$-cycle 2 in TCE-d_2 (110 °C), Figure 3A.

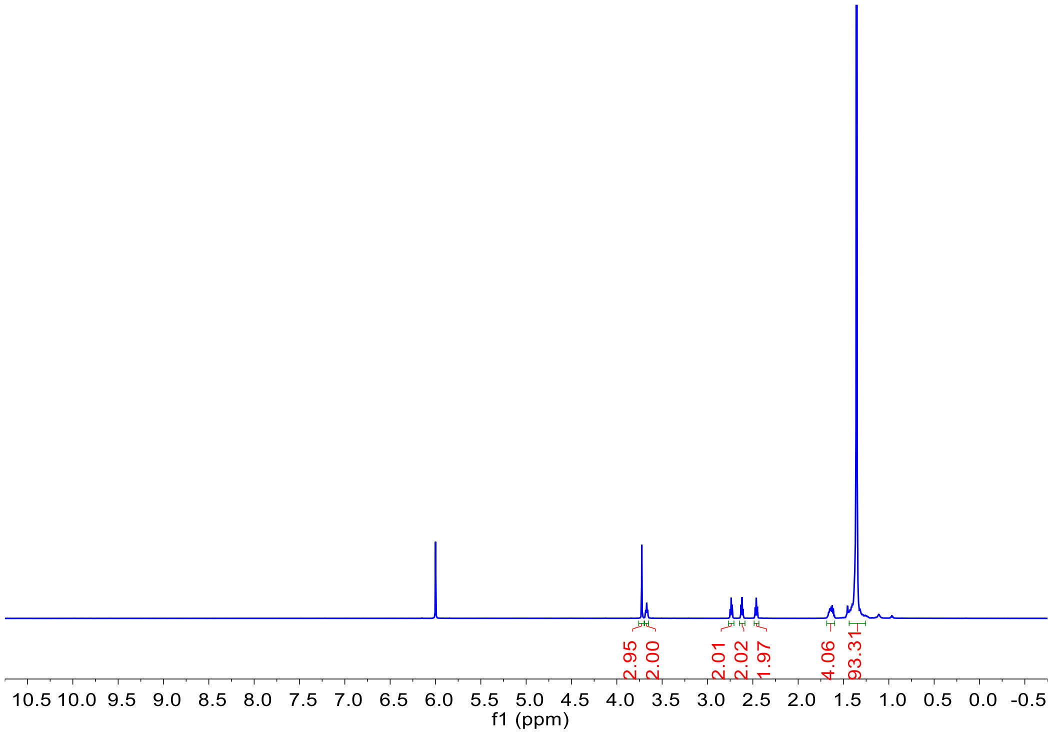

Supplementary Figure 26. ^{1}H NMR of the recovered *t*PO_0-1 from methanolysis of *r*$OBC_{9.4}$ in TCE-d_2 (110 °C), Figure 3C.

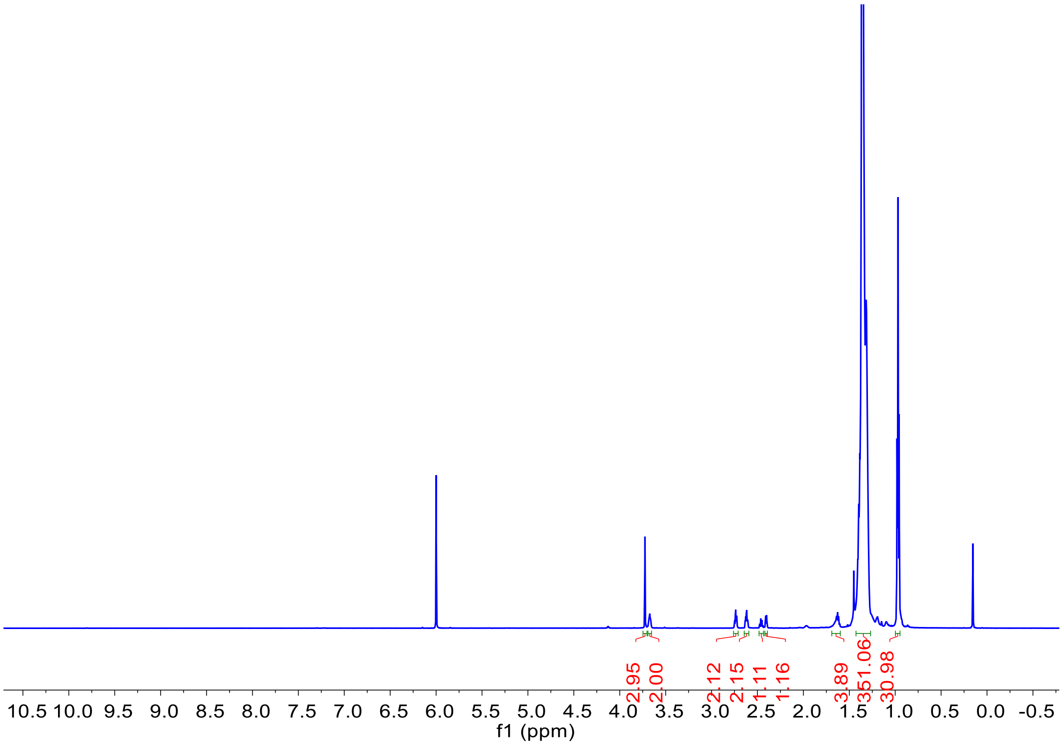

Supplementary Figure 27. ^{1}H NMR of the recovered *t*$PO_{14.8}$-1 from methanolysis of *r*$OBC_{9.4}$ in TCE-d_2 (110 °C), Figure 3C.

GPC characterizations of *t*POs, *r*POs, *r*OBCs

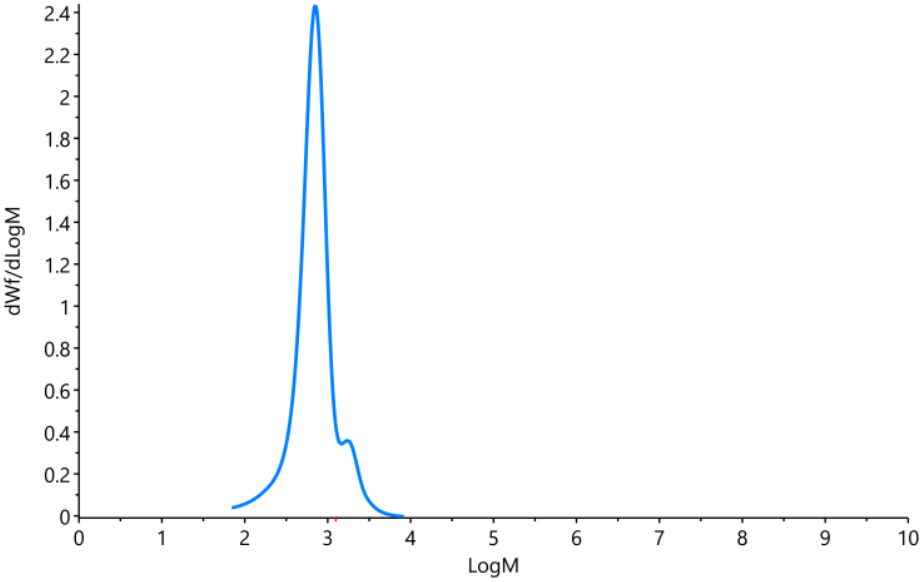

Supplementary Figure 28. GPC trace of *t*PO_0-1, Table 1 entry 1 (The shoulder peak likely arises from the esterification of the chain-end -OH and -CO_2R groups during the sample preparation and measurement).

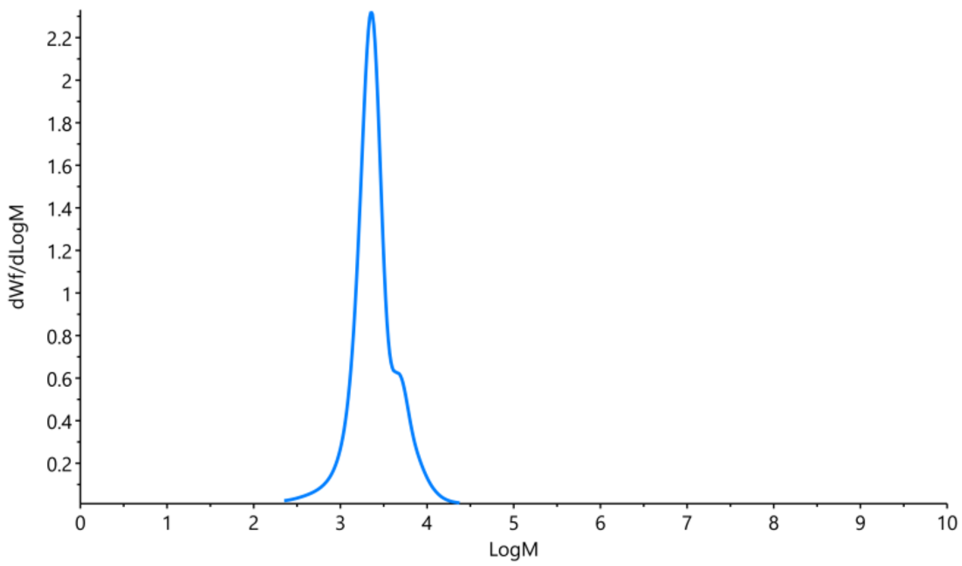

Supplementary Figure 29. GPC trace of *t*PO_0-2, Table 1 entry 2.

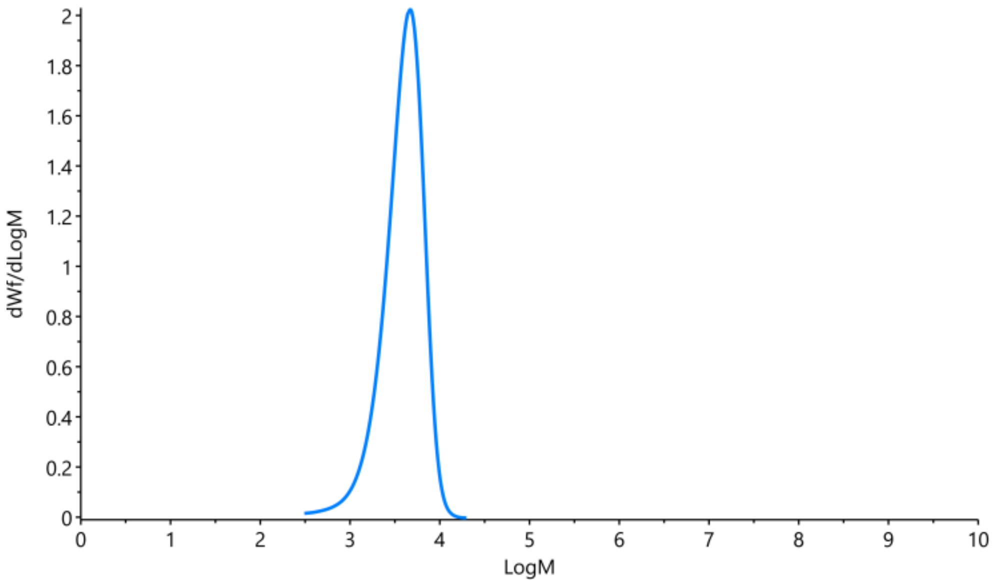

Supplementary Figure 30. GPC trace of *t*$PO_{3.1}$, Table 1 entry 3.

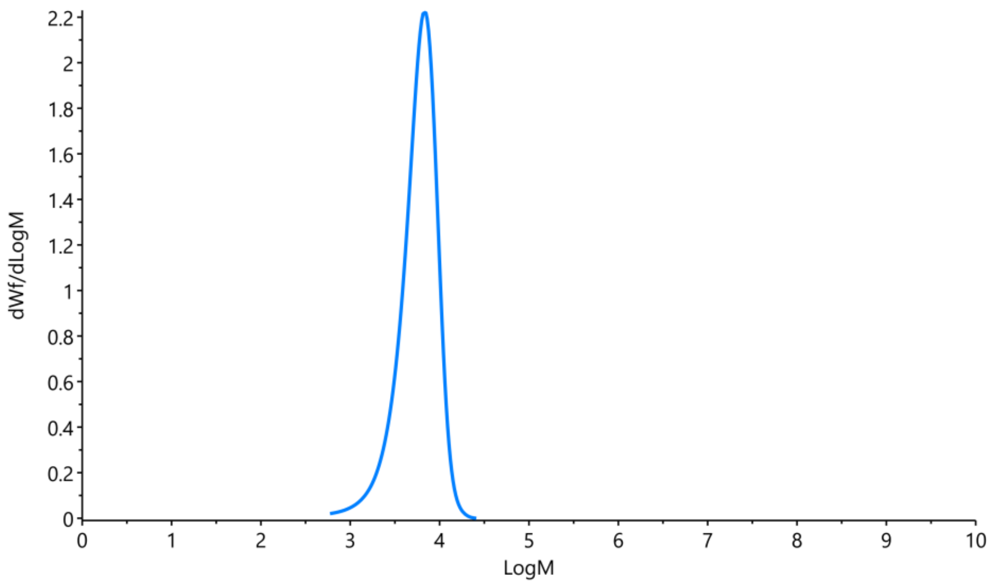

Supplementary Figure 31. GPC trace of *t*$PO_{9.6}$, Table 1 entry 4.

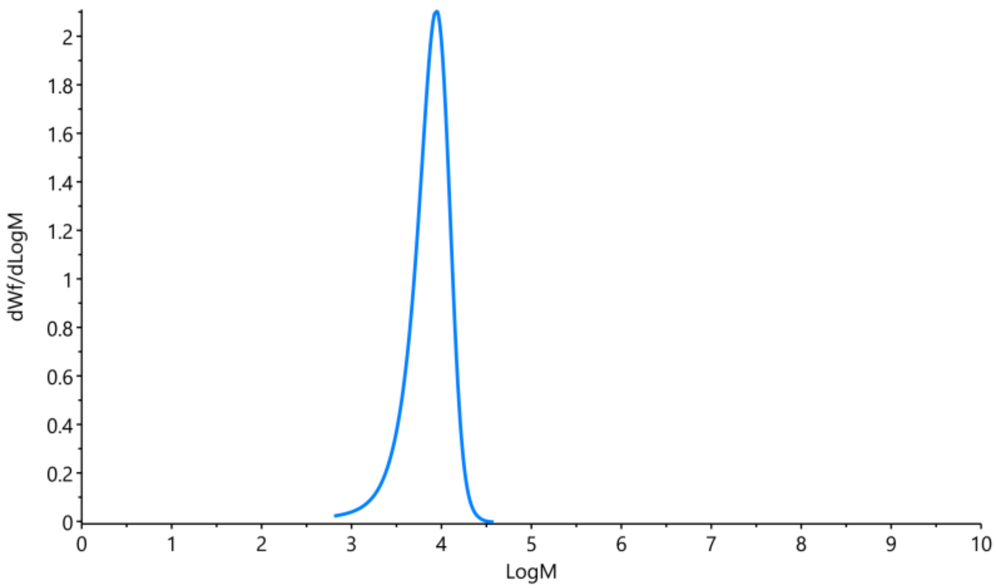

Supplementary Figure 32. GPC trace of *t*$PO_{8.9}$, Table 1 entry 5.

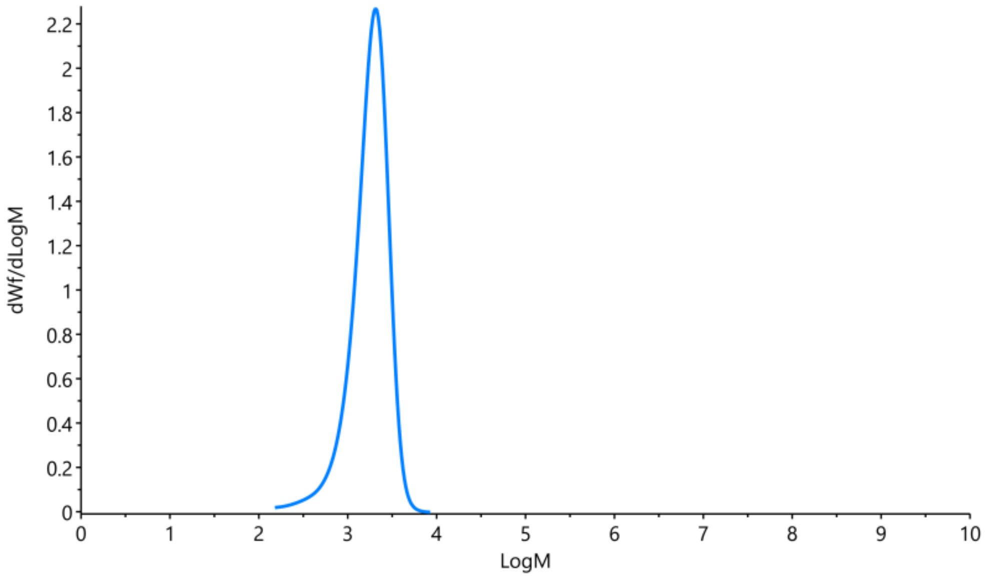

Supplementary Figure 33. GPC trace of *t*$PO_{14.8}$, Table 1 entry 6.

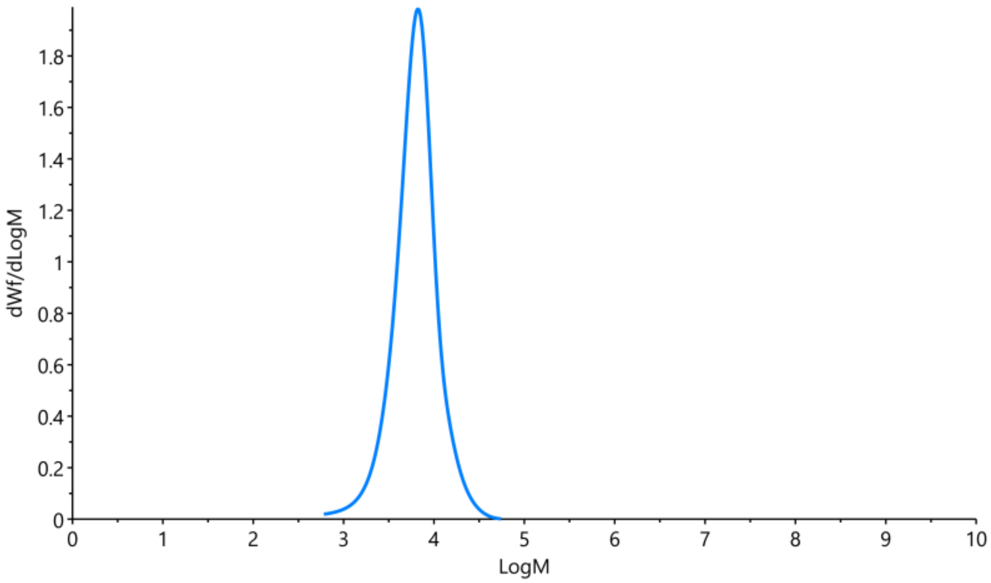

Supplementary Figure 34. GPC trace of *t*$PO_{12.2}$, Table 1 entry 7.

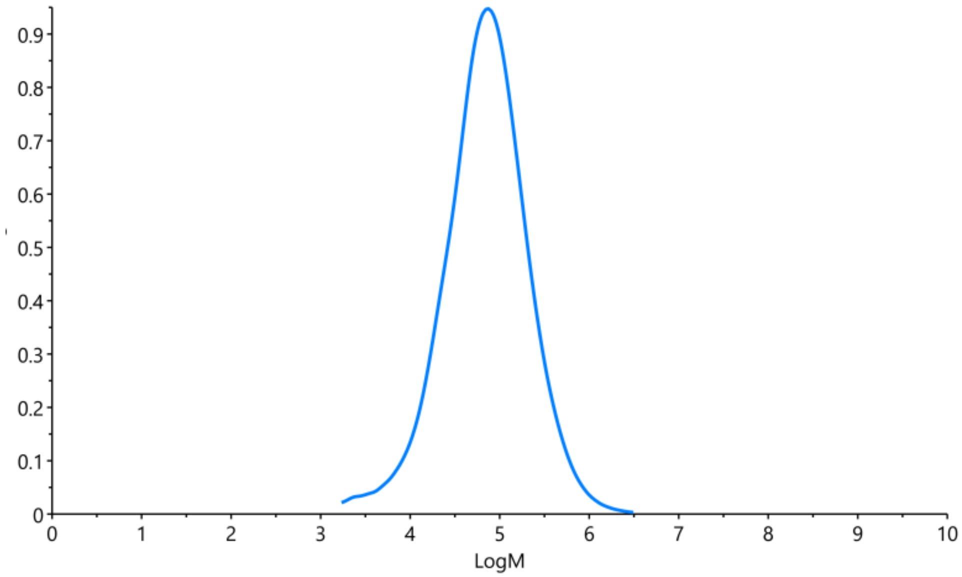

Supplementary Figure 35. GPC trace of *r*PO_0-1, Table 2 entry 1.

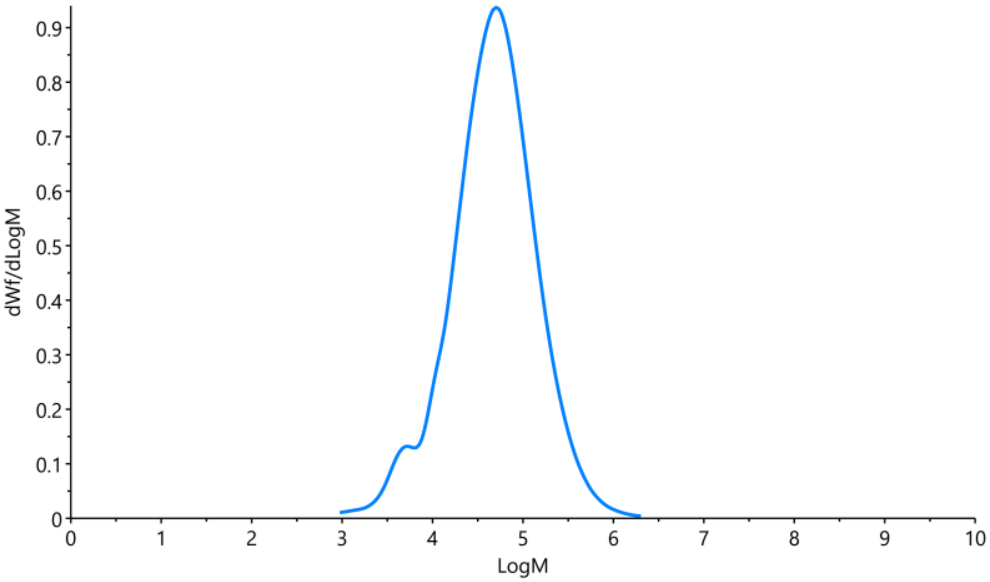

Supplementary Figure 36. GPC trace of *r*PO_0-2, Table 2 entry 2.

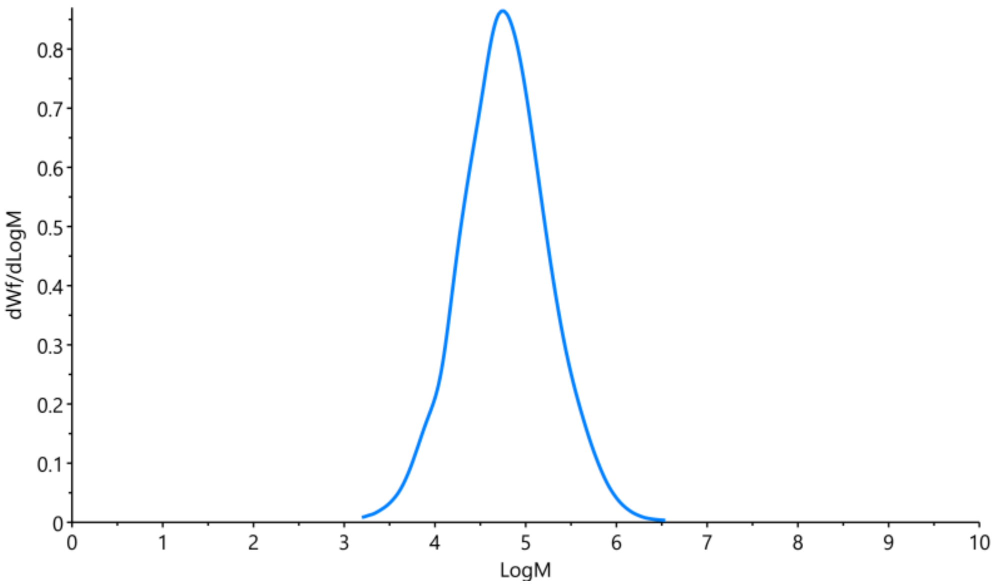

Supplementary Figure 37. GPC trace of *r*$PO_{3.1}$, Table 2 entry 3.

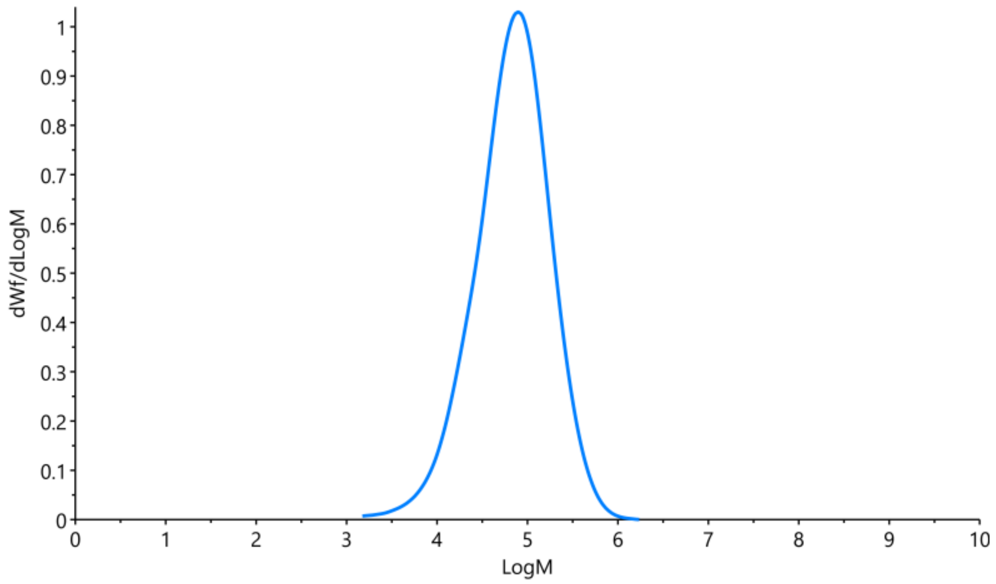

Supplementary Figure 38. GPC trace of *r*$PO_{8.9}$, Table 2 entry 4.

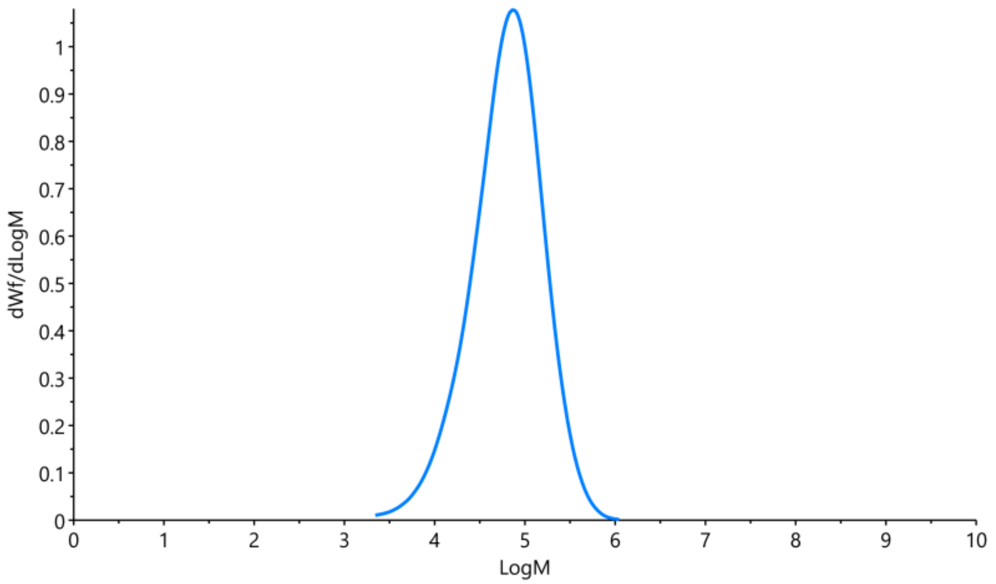

Supplementary Figure 39. GPC trace of *r*$PO_{9.6}$, Table 2 entry 5.

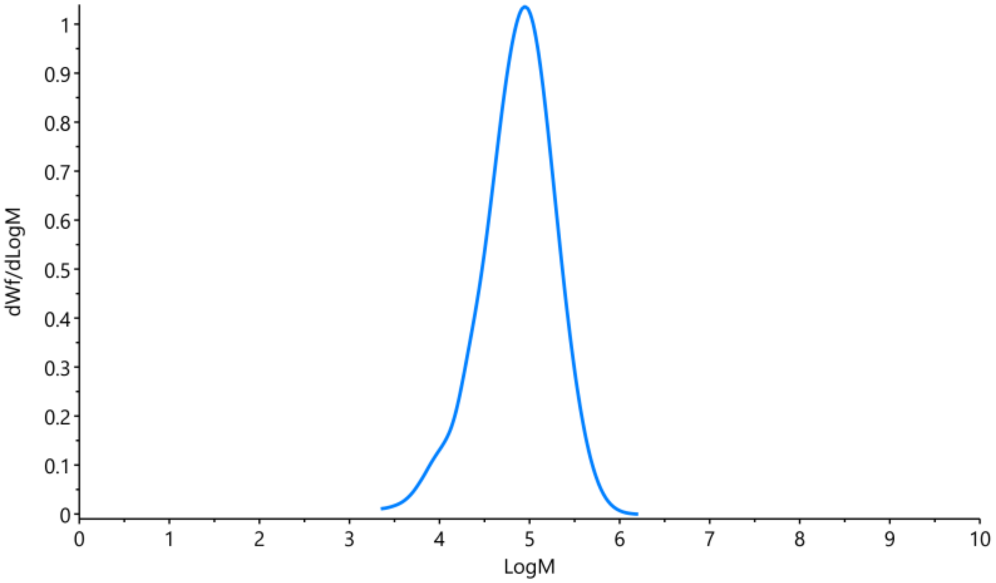

Supplementary Figure 40. GPC trace of *r*$PO_{12.2}$, Table 2 entry 6.

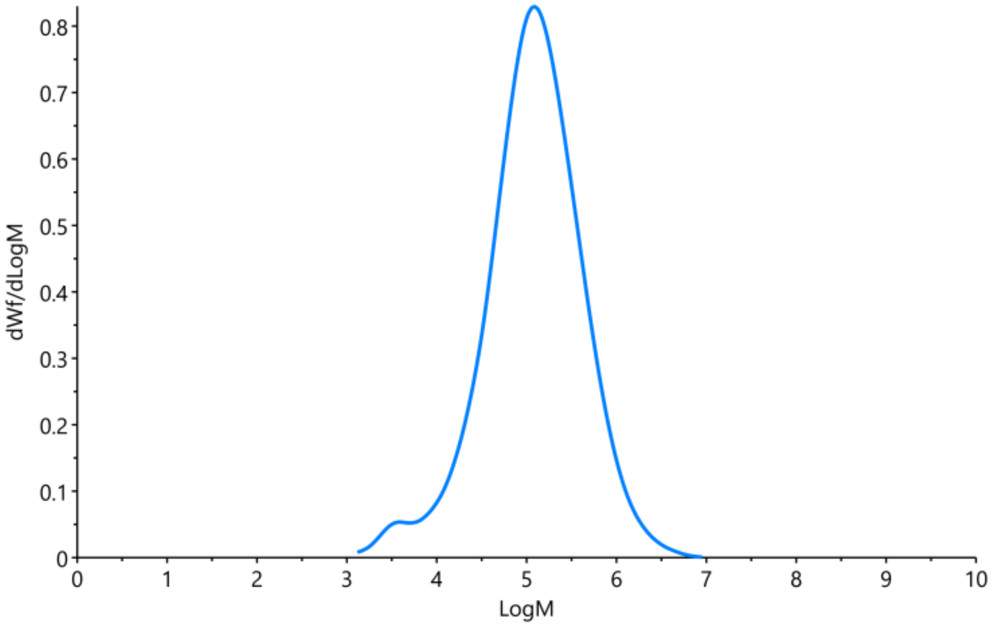

Supplementary Figure 41. GPC trace of *r*$PO_{14.8}$, Table 2 entry 7.

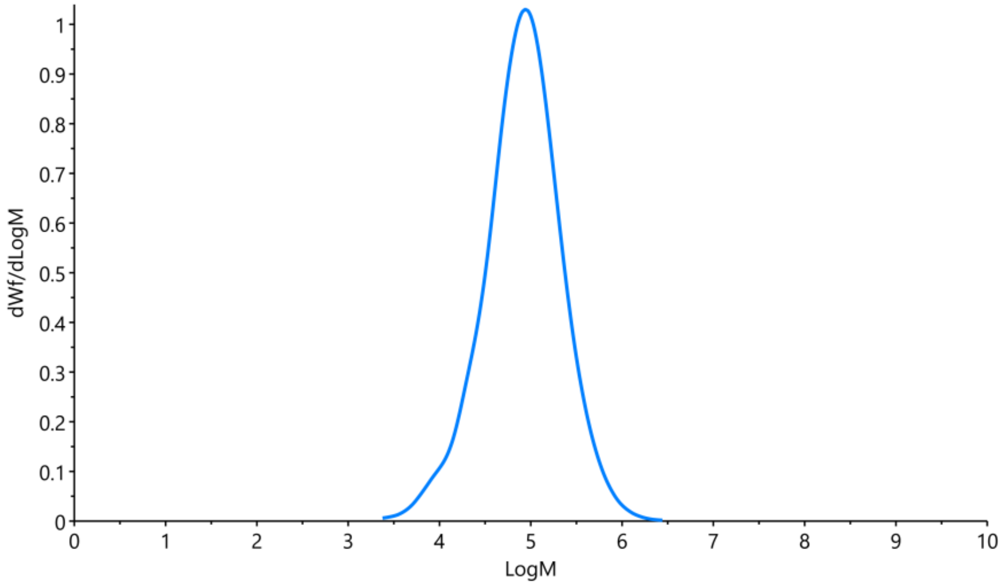

Supplementary Figure 42. GPC trace of *r*$OBC_{7.7}$, Table 2 entry 8.

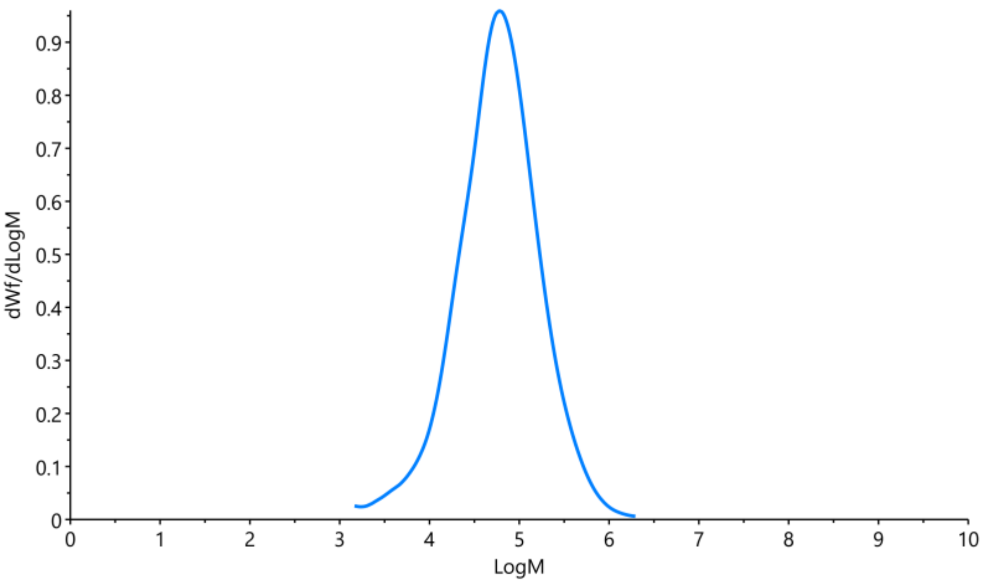

Supplementary Figure 43. GPC trace of *r*OBC$_{9.4}$, Table 2 entry 9.

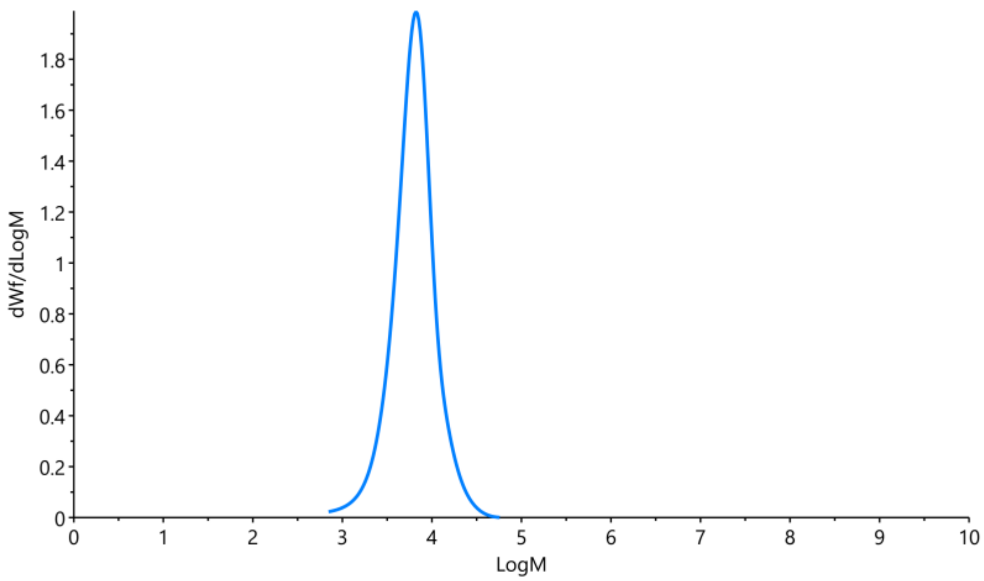

Supplementary Figure 44. GPC trace of the recovered *t*PO$_{9.6}$-cycle 1, Figure 3a.

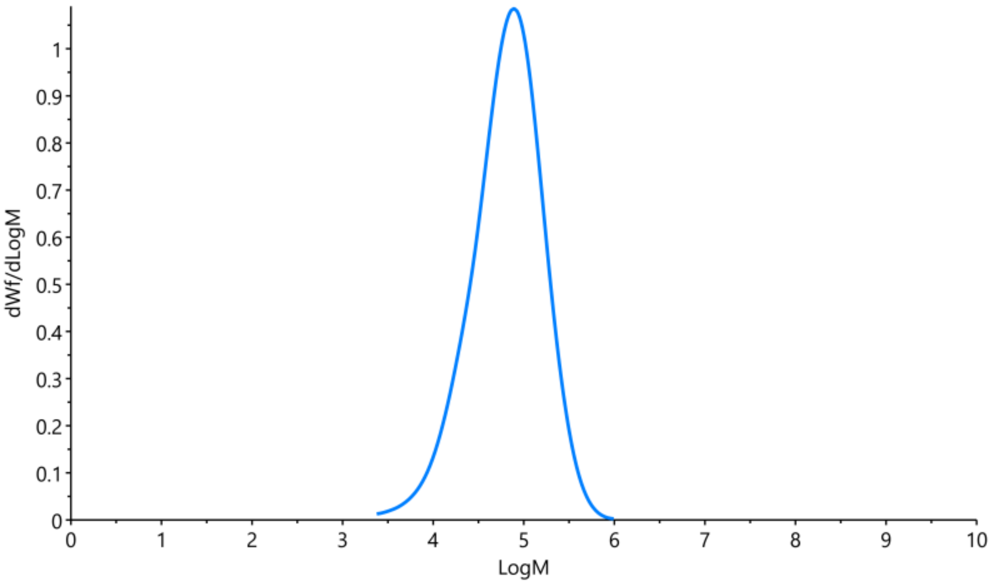

Supplementary Figure 45. GPC trace of *r*$PO_{9.6}$-cycle 1, Figure 3a.

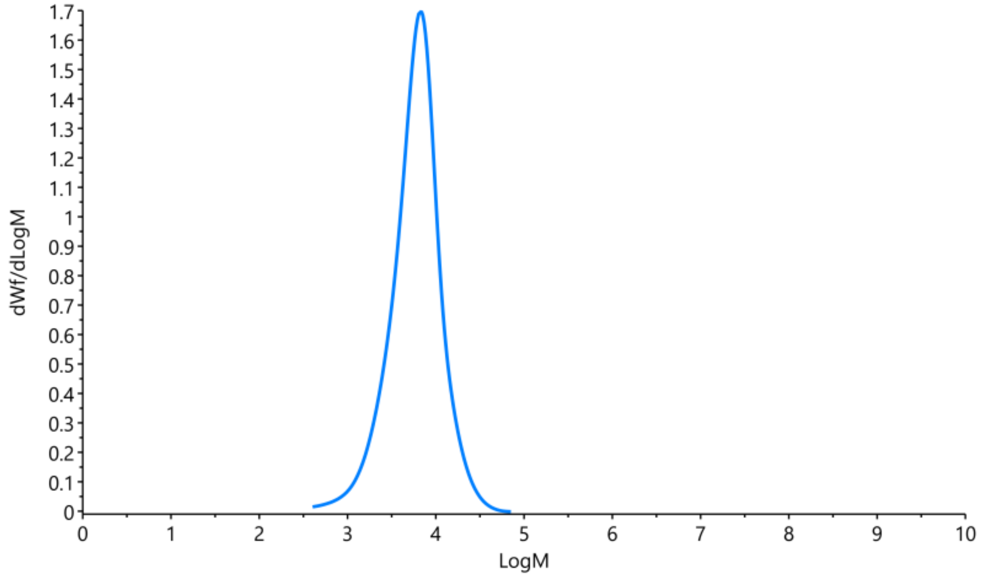

Supplementary Figure 46. GPC trace of the recovered *t*$PO_{9.6}$-cycle 2, Figure 3a.

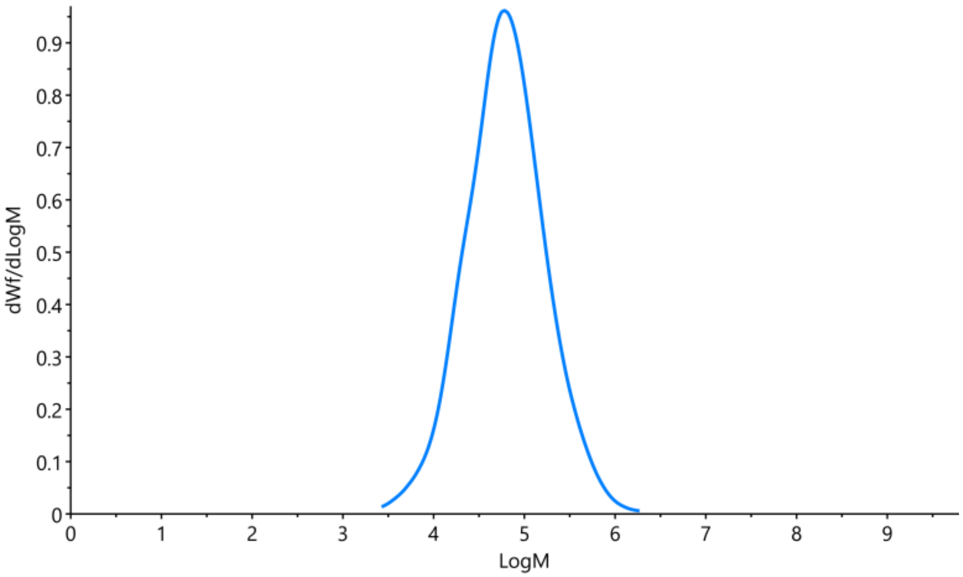

Supplementary Figure 47. GPC trace of *r*$PO_{9.6}$-cycle 2, Figure 3a.

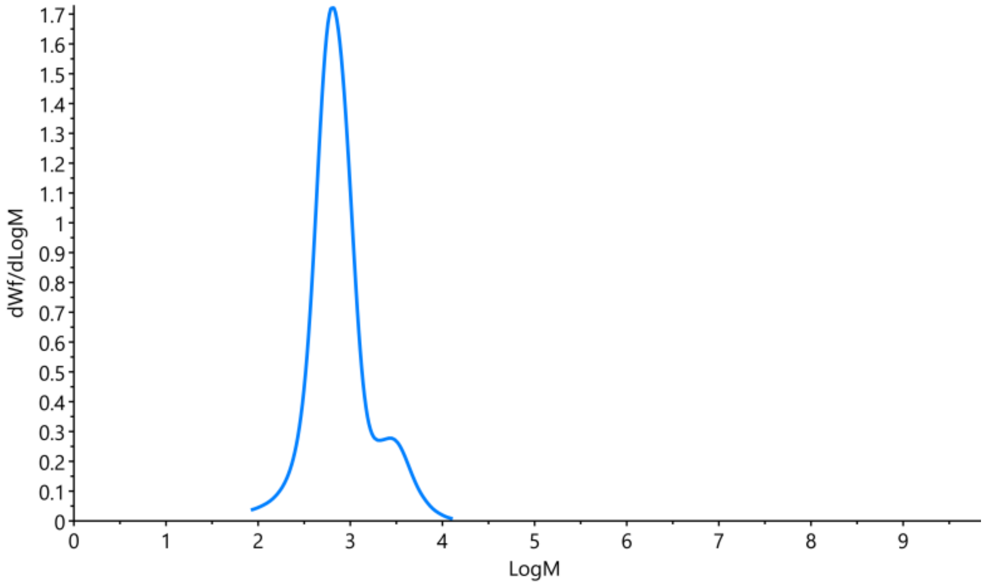

Supplementary Figure 48. GPC trace of the recovered *t*PO_0-1 from methanolysis of *r*$OBC_{9.4}$, Figure 3c.

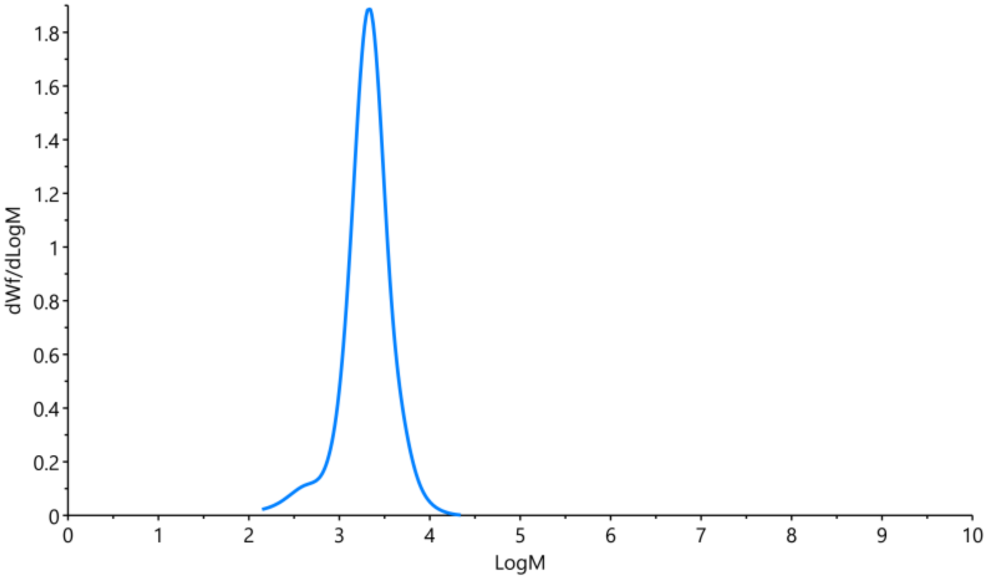

Supplementary Figure 49. GPC trace of the recovered *t*PO$_{14.8}$-1 from methanolysis of *r*OBC$_{9.4}$, Figure 3c.

MALDI-TOF characterizations of *t*POs

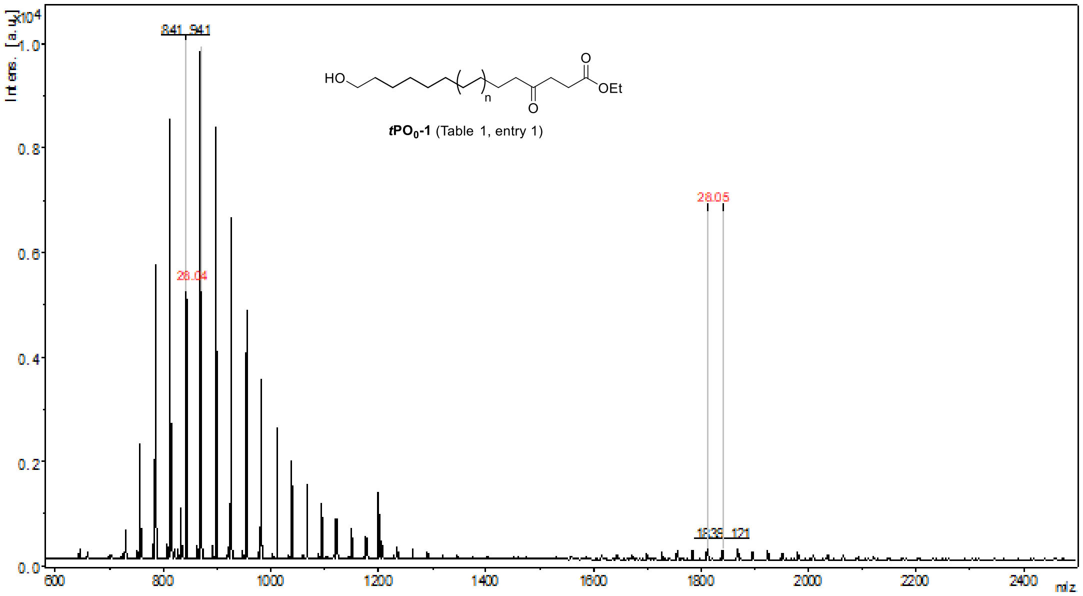

Supplementary Figure 50. MALDI-TOF mass spectrum of *t*PO$_0$-1, Table 1 entry 1.

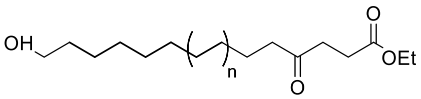

***t*PO$_0$-2** (Table 1, entry 2)

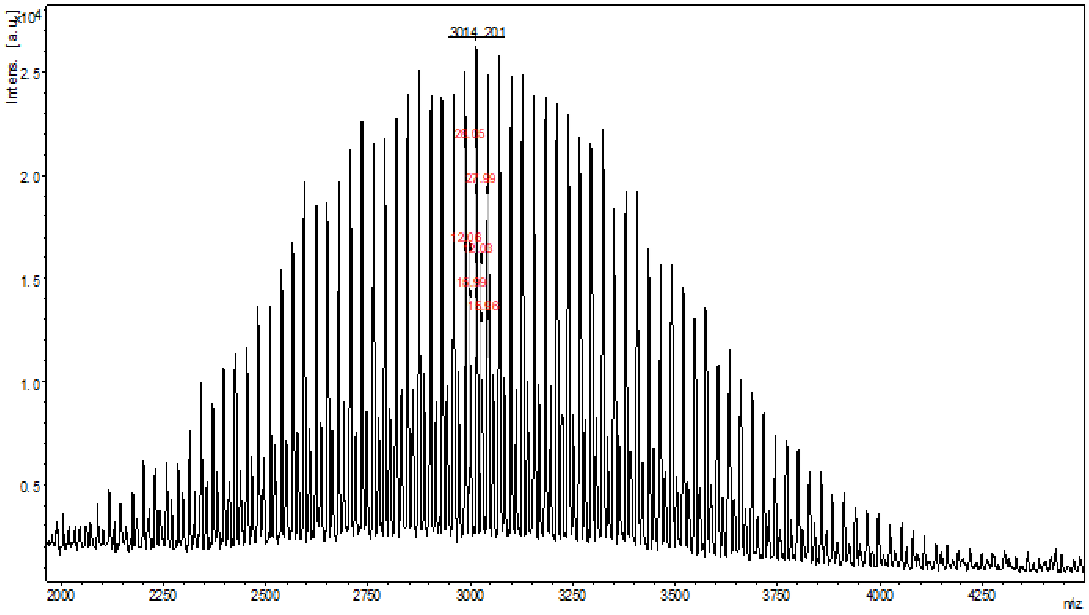

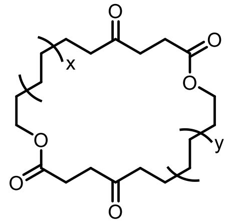

cyclic dimer

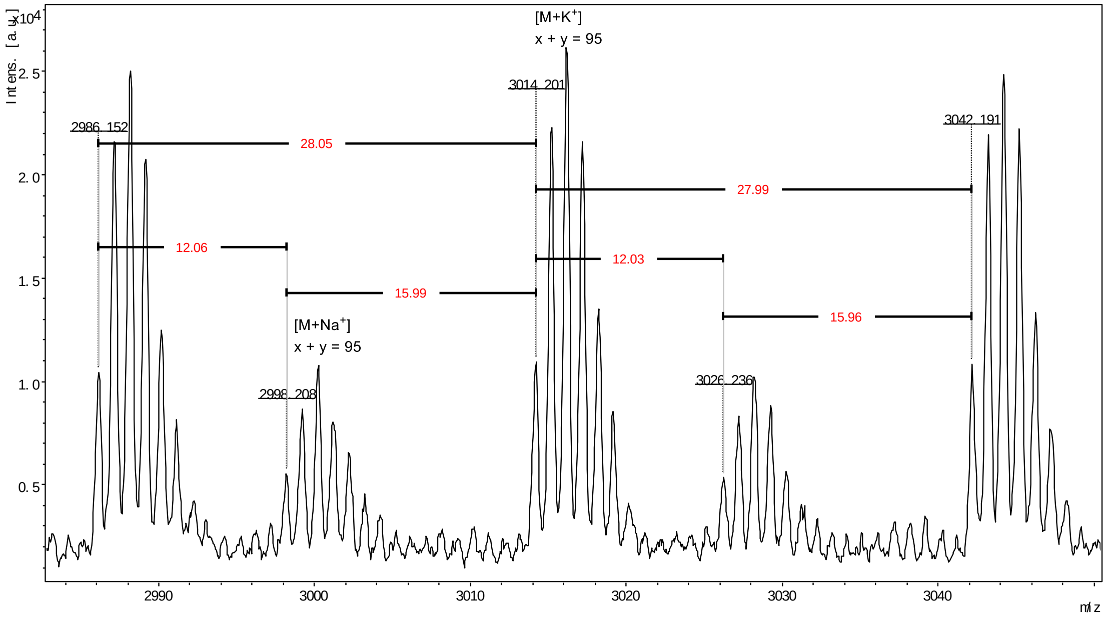

Supplementary Figure 51. MALDI-TOF mass spectrum of *t*PO_0-2, Table 1 entry 2.

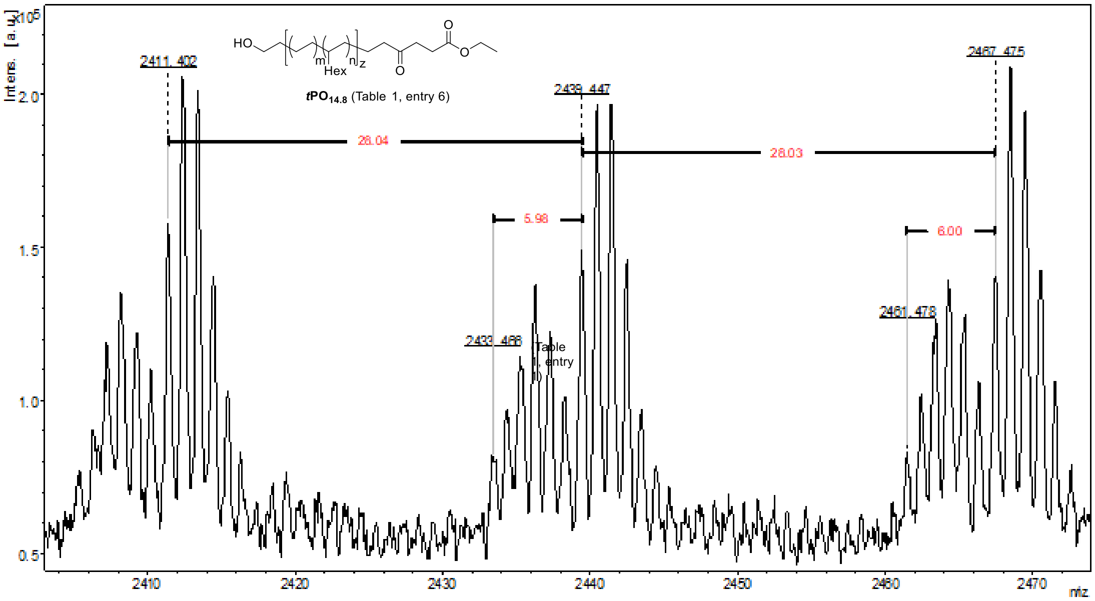

Supplementary Figure 52. MALDI-TOF mass spectrum of *t*$PO_{14.8}$, Table 1 entry 6.

TGA measurements of *r*PO_0-1, *r*$PO_{3.1}$, *r*$PO_{9.6}$, *r*$PO_{8.9}$, *r*$OBC_{7.7}$ and *c*$PO_{11.2}$

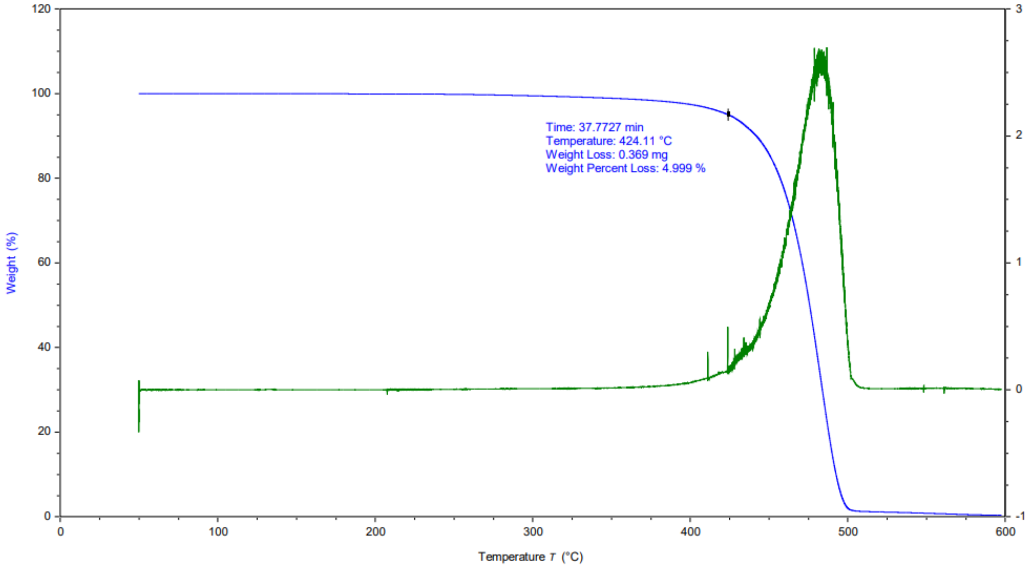

Supplementary Figure 53. TGA trace of *r*PO_0-1 under nitrogen atmosphere, Table 2 entry 1.

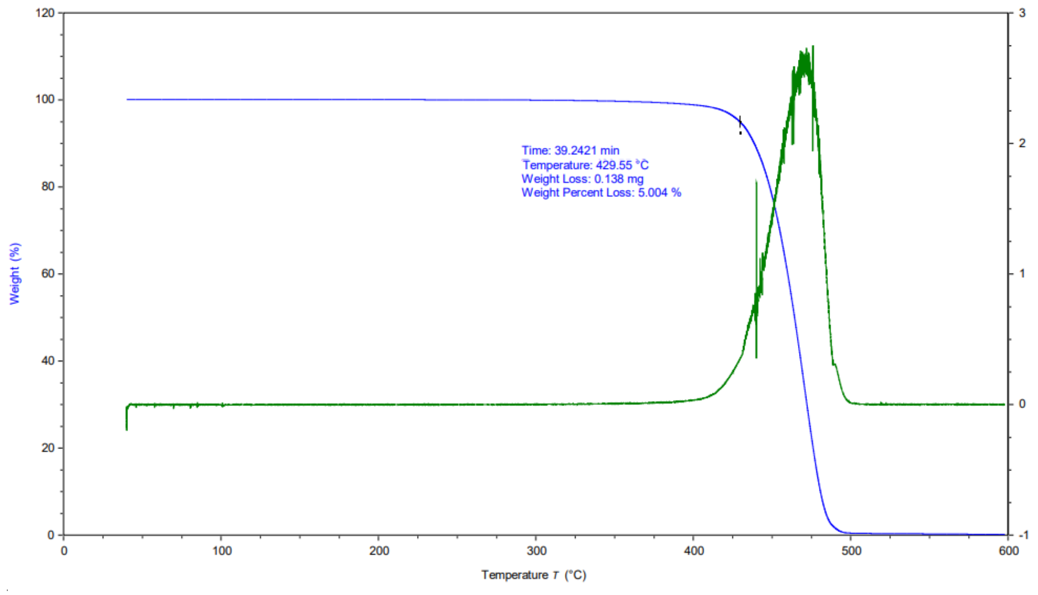

Supplementary Figure 54. TGA trace of *r*$PO_{3.1}$ under nitrogen atmosphere, Table 2 entry 3.

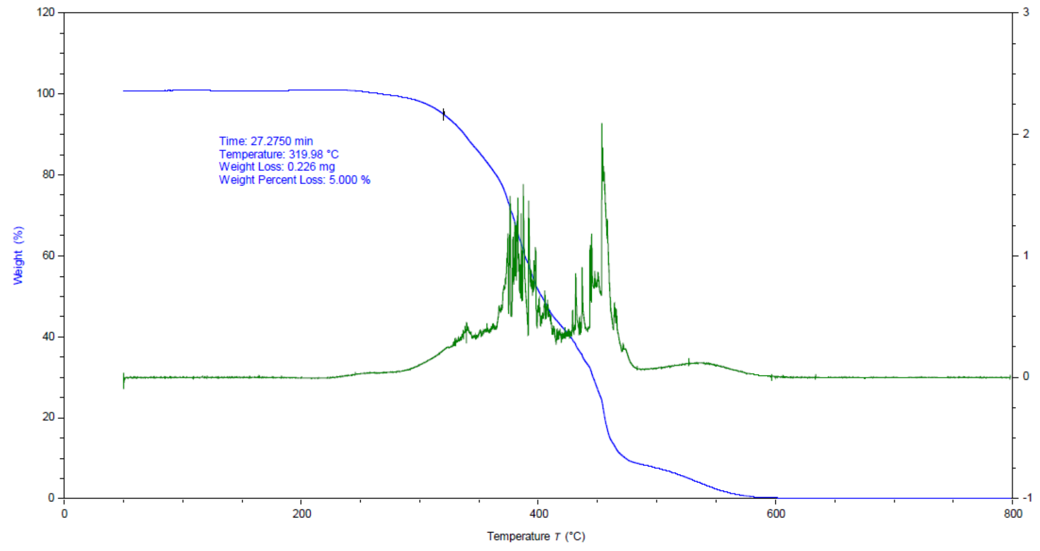

Supplementary Figure 55. TGA trace of *r*$PO_{3.1}$ under air condition, Table 2 entry 3.

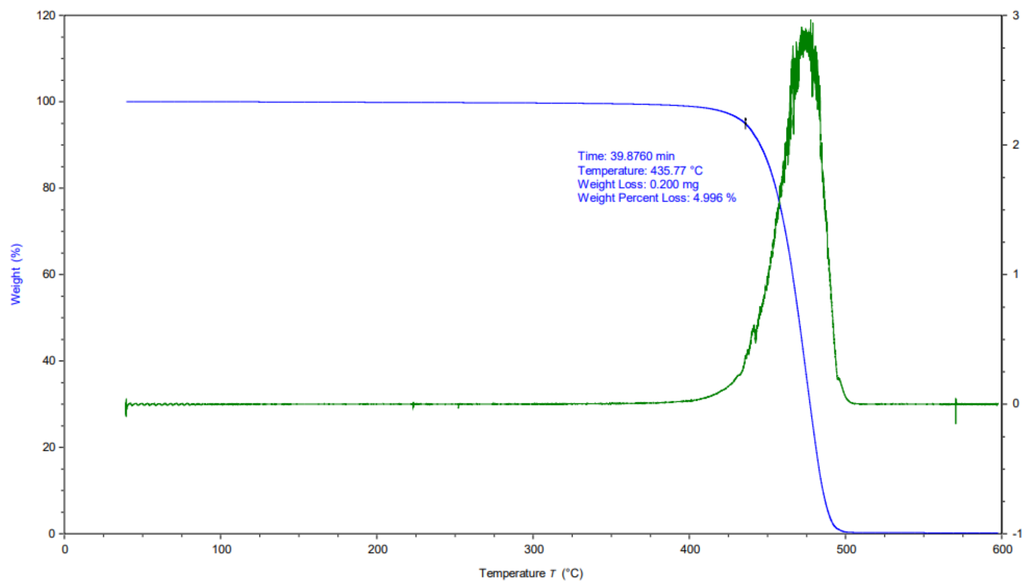

Supplementary Figure 56. TGA trace of *r*$PO_{8.9}$ under nitrogen atmosphere, Table 2 entry 4.

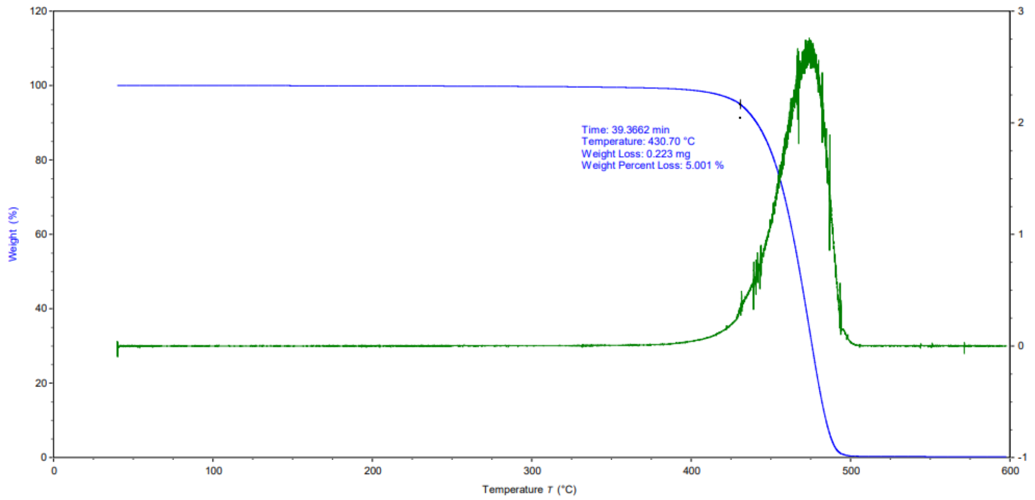

Supplementary Figure 57. TGA trace of *r*$PO_{9.6}$ under nitrogen atmosphere, Table 2 entry 5.

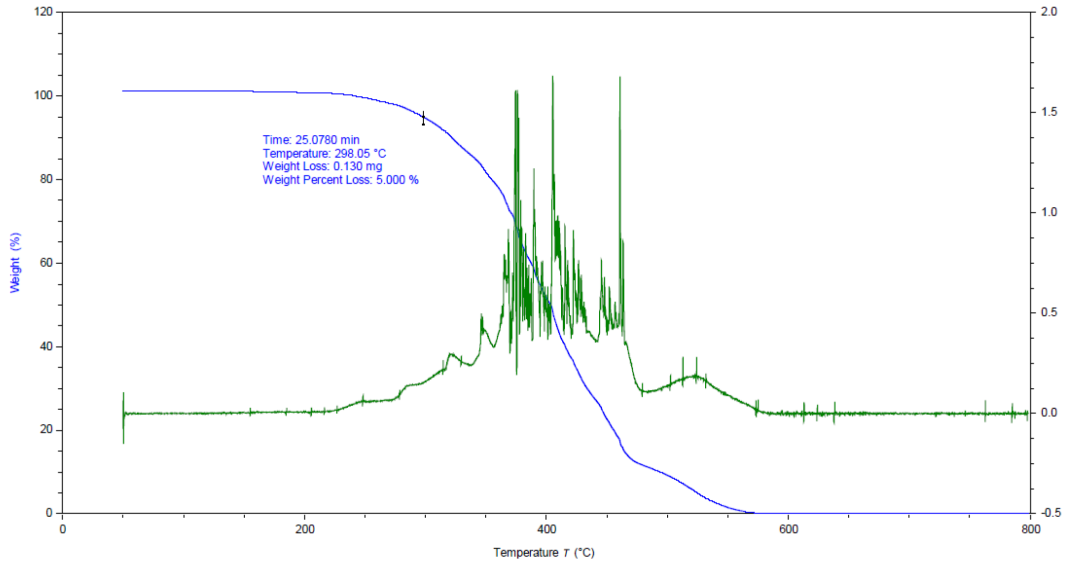

Supplementary Figure 58. TGA trace of *r*$PO_{9.6}$ under air condition, Table 2 entry 5.

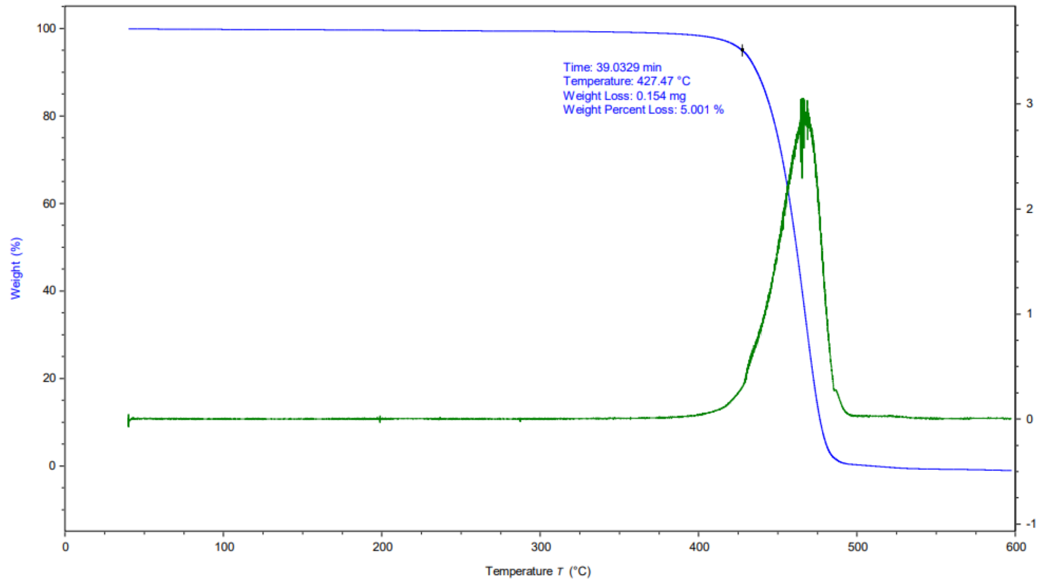

Supplementary Figure 59. TGA trace of *r*$OBC_{7.7}$ under nitrogen atmosphere, Table 2 entry 9.

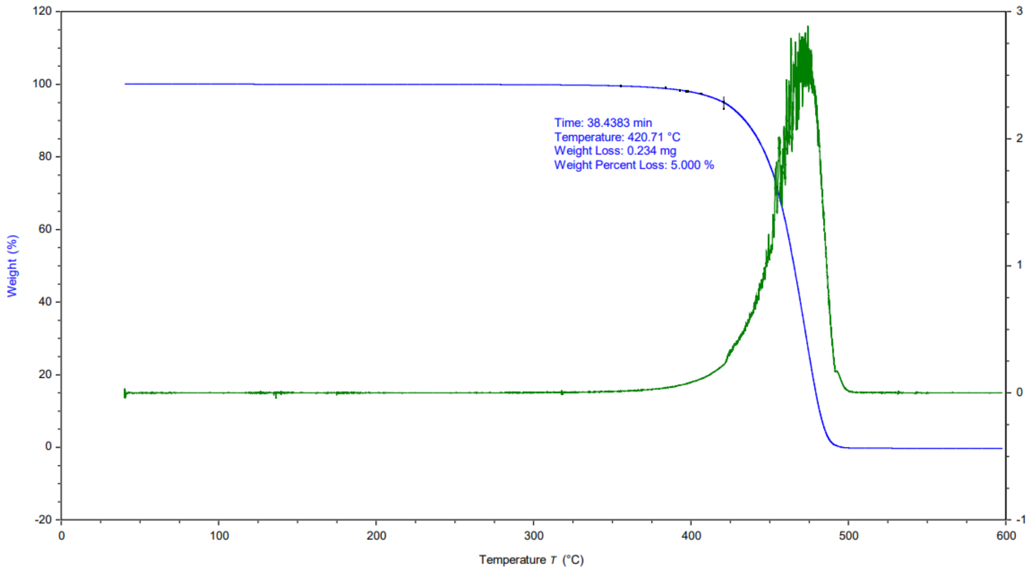

Supplementary Figure 60. TGA trace of *c*$PO_{11.2}$ under nitrogen atmosphere.

DSC characterizations of *r*POs and *r*OBCs

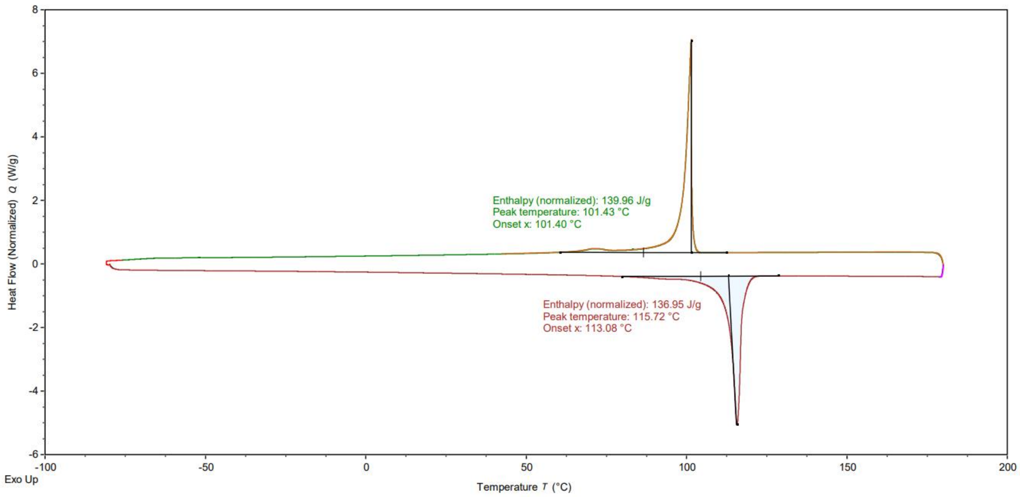

Supplementary Figure 61. DSC trace of *r*PO_0-1, Table 2 entry 1.

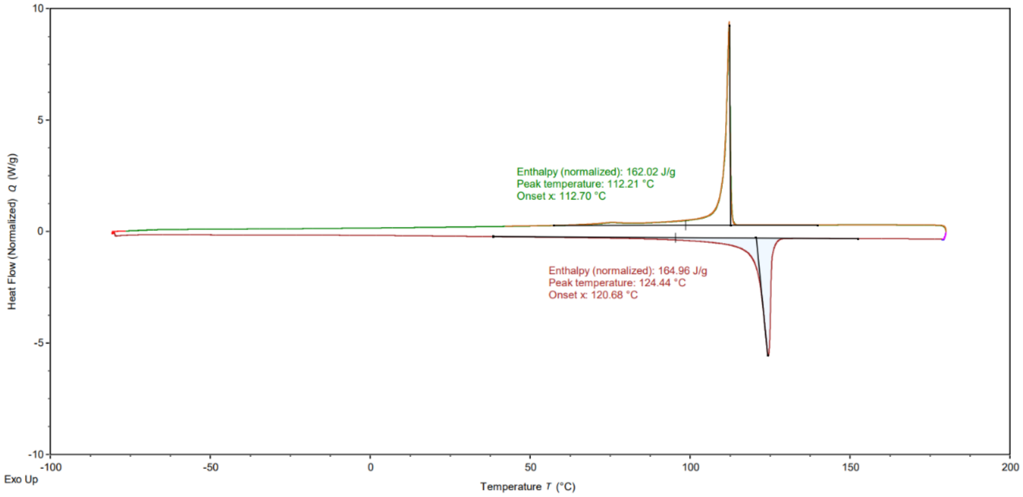

Supplementary Figure 62. DSC trace of *r*PO_0-2, Table 2 entry 2.

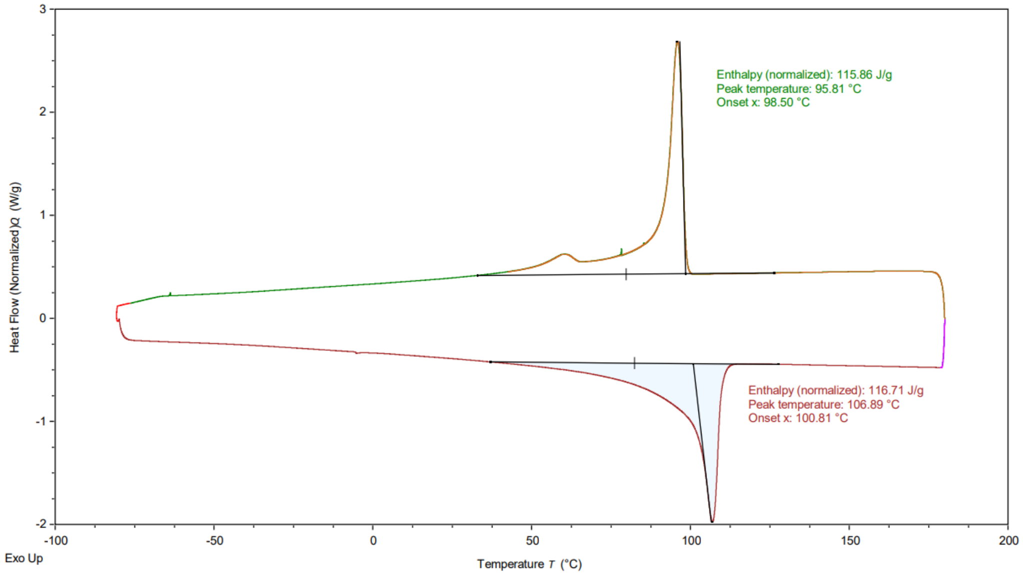

Supplementary Figure 63. DSC trace of *r*$PO_{3.1}$, Table 2 entry 3.

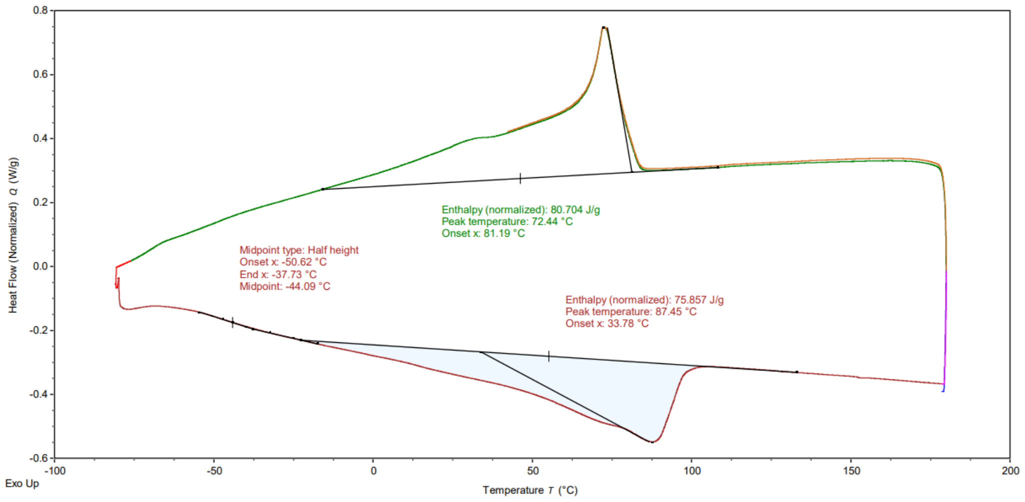

Supplementary Figure 64. DSC trace of *r*PO$_{8.9}$, Table 2 entry 4.

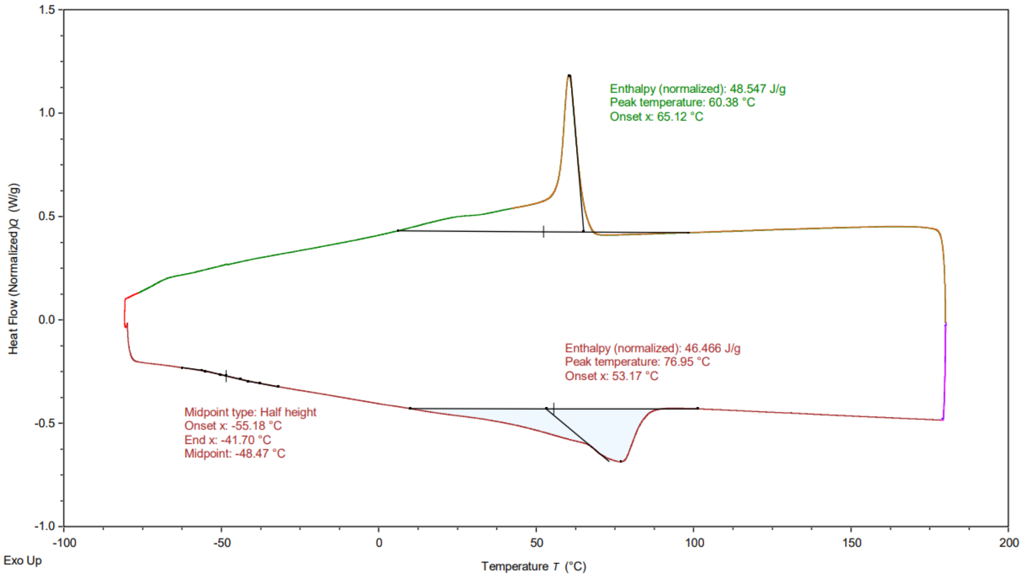

Supplementary Figure 65. DSC trace of *r*PO$_{9.6}$, Table 2 entry 5.

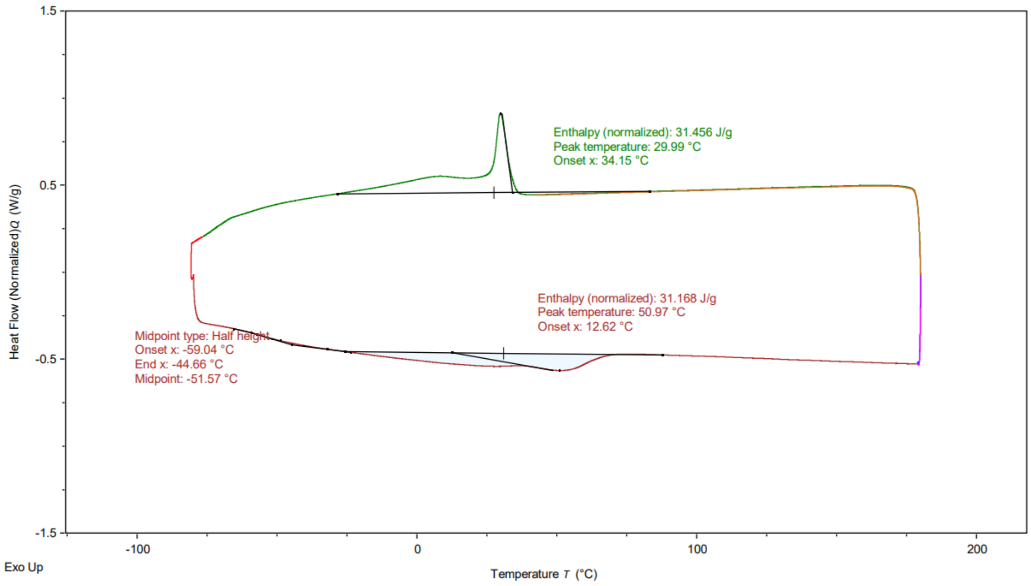

Supplementary Figure 66. DSC trace of *r*$PO_{12.2}$, Table 2 entry 6.

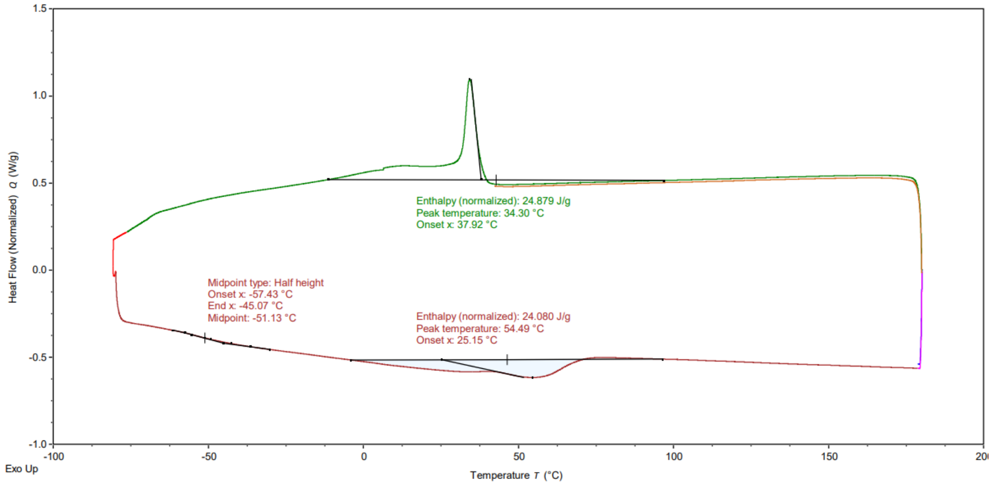

Supplementary Figure 67. DSC trace of *r*$PO_{14.8}$, Table 2 entry 7.

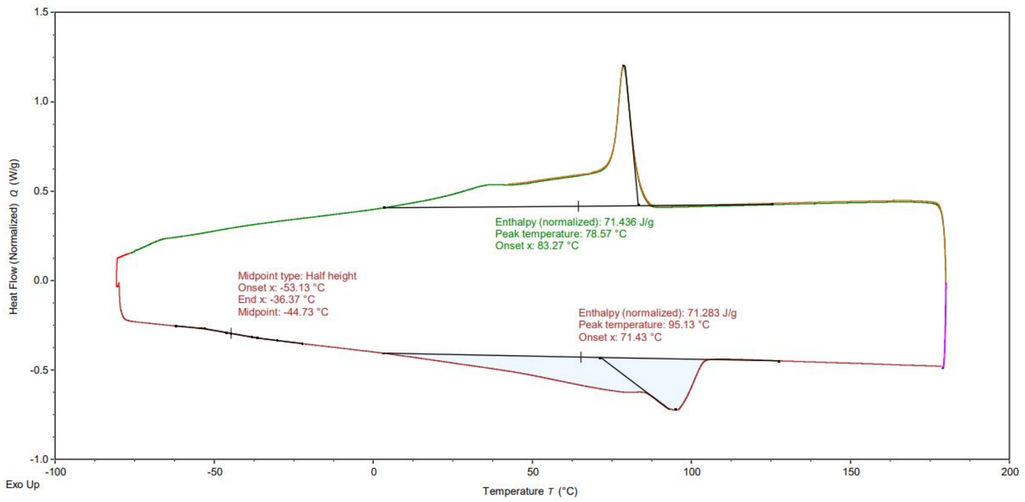

Supplementary Figure 68. DSC trace of *r*OBC$_{7.7}$, Table 2 entry 8.

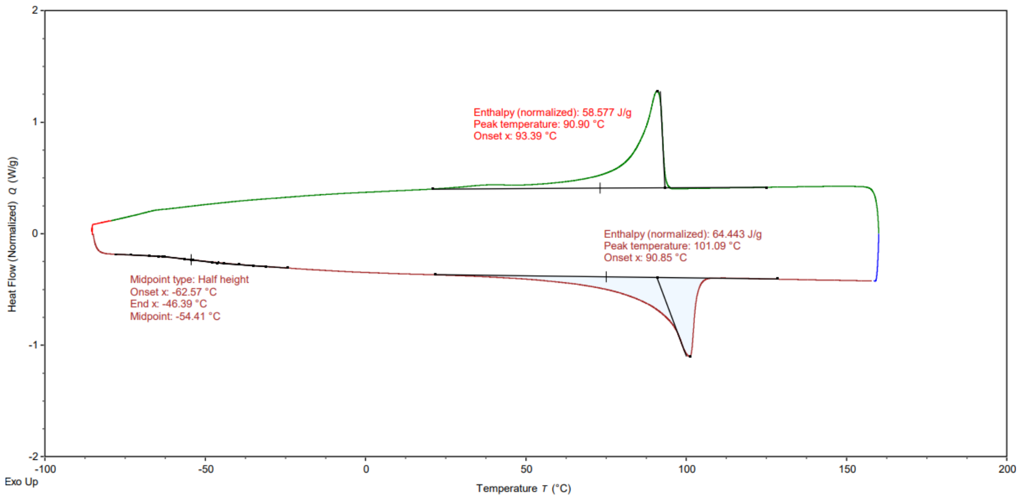

Supplementary Figure 69. DSC trace of *r*OBC$_{9.4}$, Table 2 entry 9.

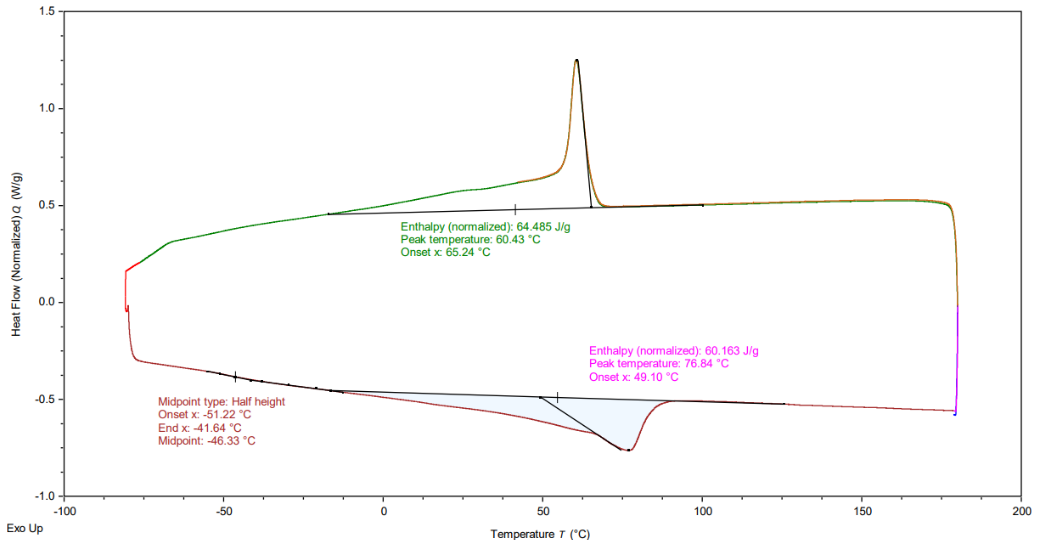

Supplementary Figure 70 DSC trace of *r*PO$_{9.6}$ -recyle 1, Figure 3A.

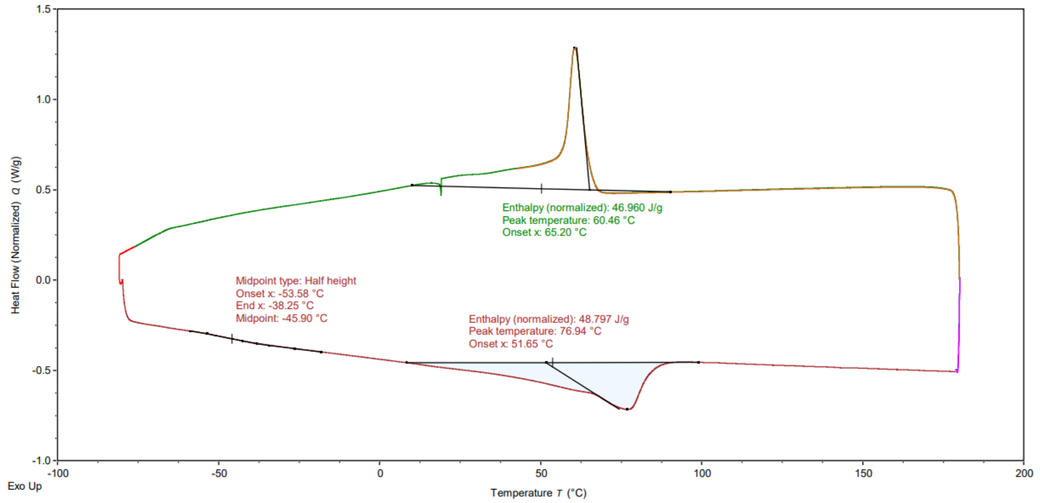

Supplementary Figure 71. DSC trace of *r*PO$_{9.6}$ -recyle 2, Figure 3A.

Powder X-ray characterizations of *r*POs and *r*OBCs

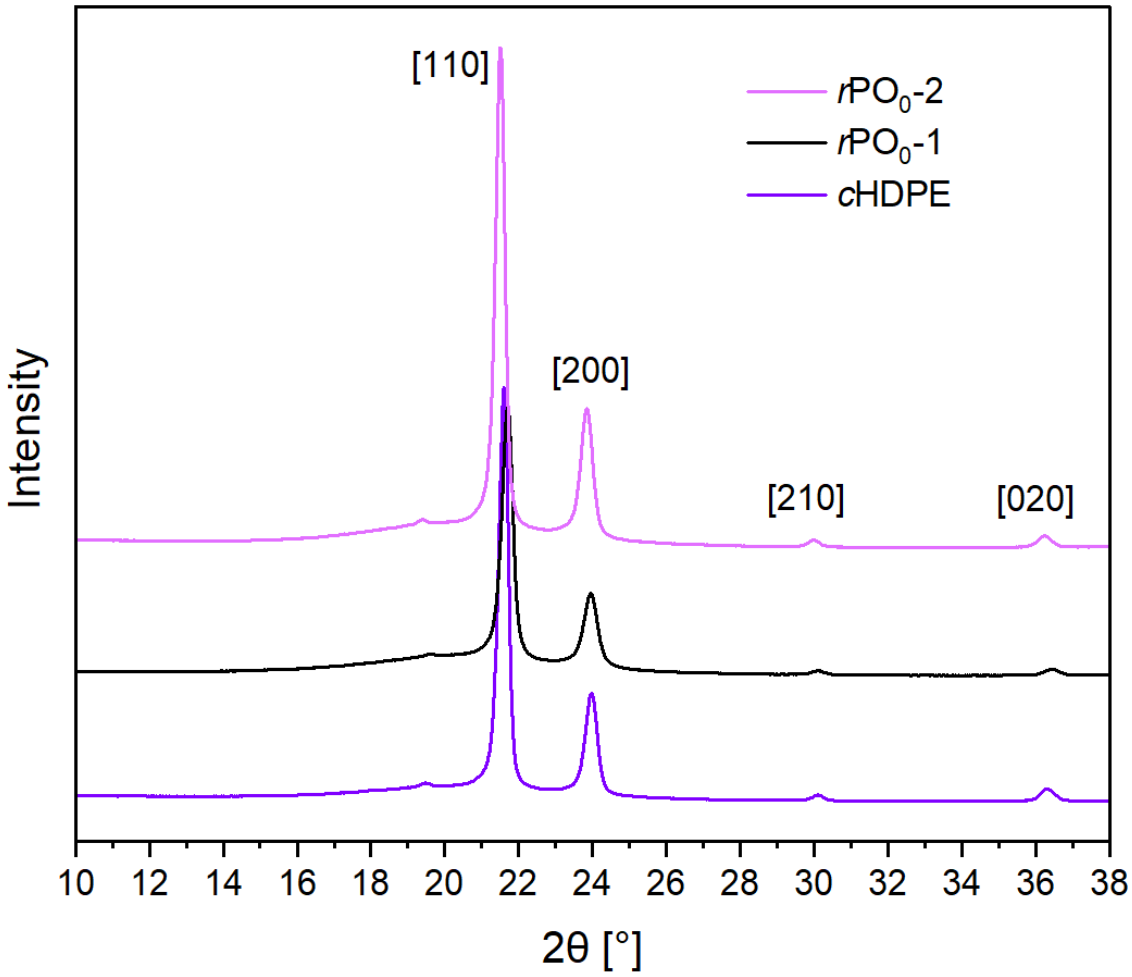

Supplementary Figure 72. XRD diffraction patterns of the commercial HDPE (DOW DMDA-8904 NT 7, *c*HDPE), *r*PO_0-1, and *r*PO_0-2 films.

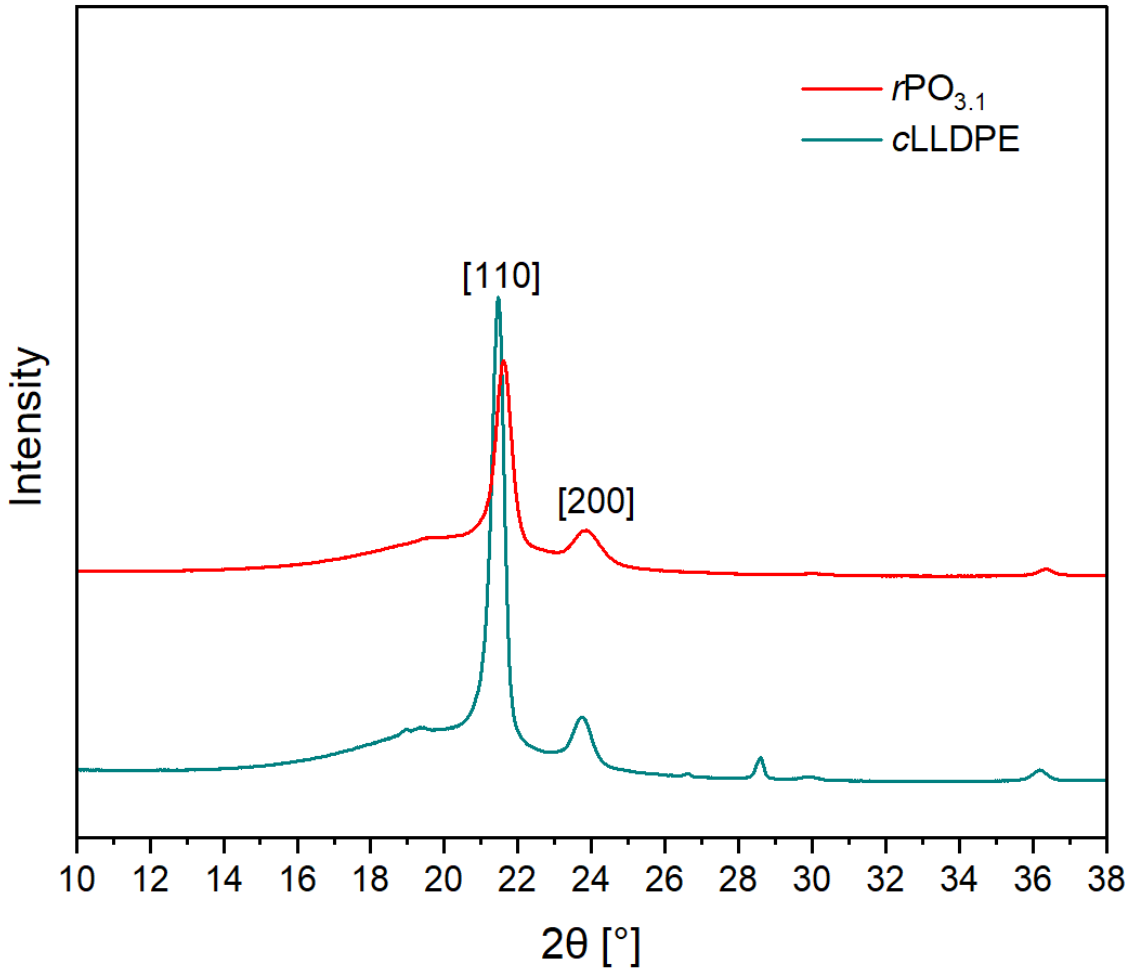

Supplementary Figure 73. XRD diffraction patterns of the commercial LLDPE (DOW TUFLIN™ HSE-1003 NT 7, *c*LLDPE) and $rPO_{3.1}$ films.

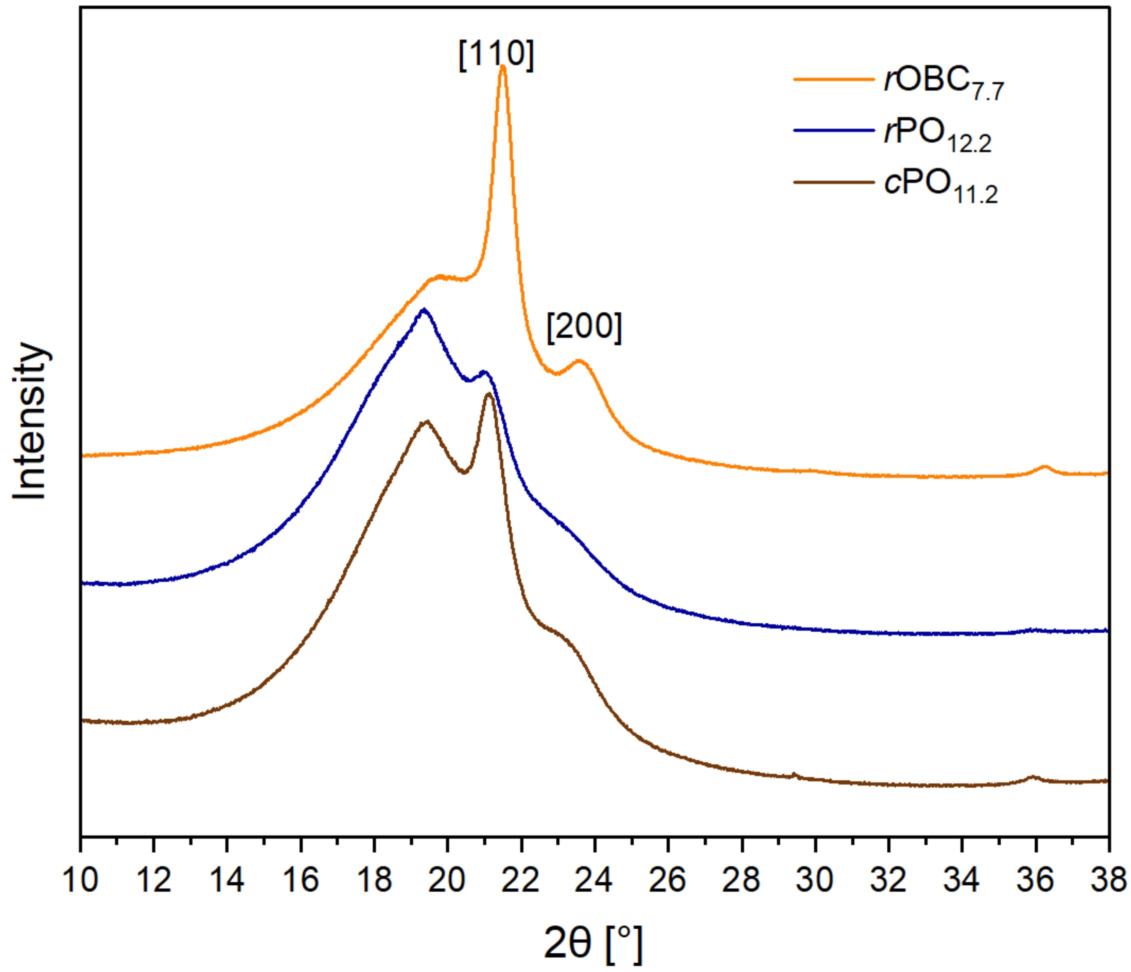

Supplementary Figure 74. XRD diffraction patterns of the commercial POE (DOW ENGAGE™ PV 8669, cPO11.2), rPO12.2, and rOBC7.7 films.

S2. Supplementary Tables

Dogbone tensile specimens and tensile test.

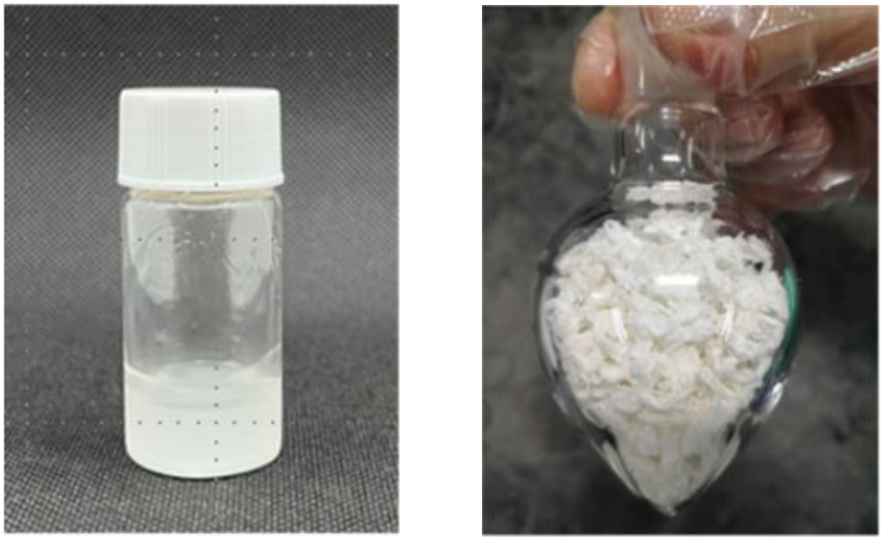

Supplementary Figure 75. Digital images of build block *t*$PO_{9.6}$ (left) and polymer *r*$PO_{9.6}$ (right)

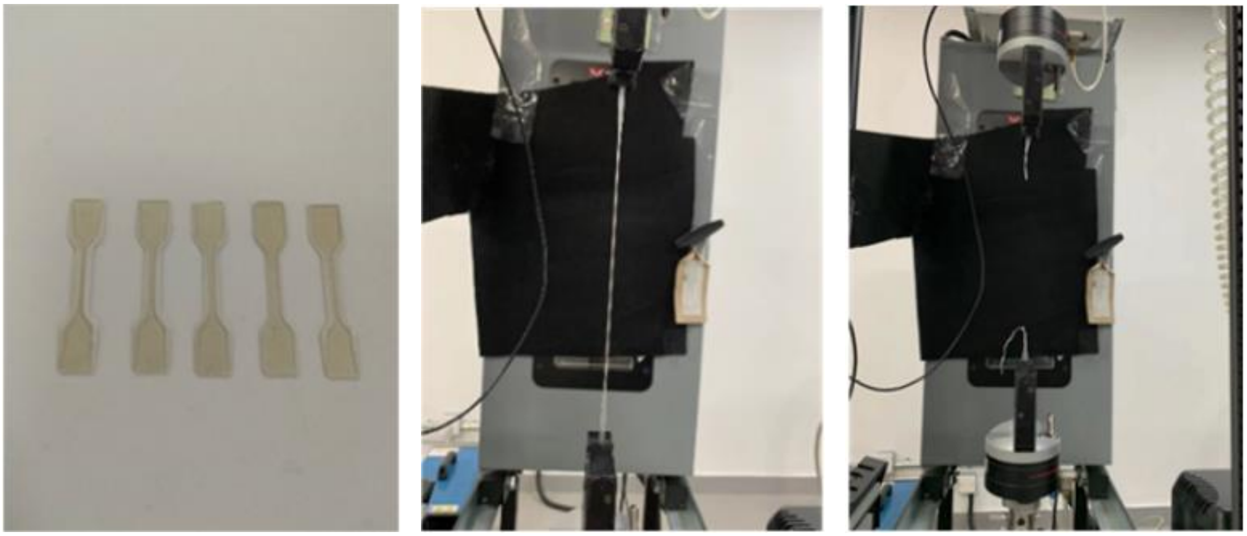

Supplementary Figure 76. Digital images of dogbone tensile specimens generated from reprocessing post-tensile test specimens (left), tensile testing of *r*$PO_{9.6}$ and specimen after break. The results were reported in Supplementary Table 1.

Supplementary Table 1. Data of tensile stress-strain testing.

Polymer	Young's modulus (MPa)	Tensile Strength at 100% Strain (σ_{100}, MPa)	Tensile Strength at Break (σ_b, MPa)	Strain at Break (ε_b, %)
*r*PO$_0$-1	742.7±73.8	20.04 ± 0.75[a]	9.59 ± 3.03	57.32 ± 25.47
*r*PO$_{3.1}$	214.2±40.5	9.22 ± 0.11	14.92 ± 1.08	994 ± 59
*r*PO$_{9.6}$	-	3.19 ± 0.13	14.0 ± 1.24	2347 ± 271
*r*PO$_{8.9}$	-	4.08 ± 0.08	17.83 ± 0.60	2333 ± 33
*r*PO$_{12.2}$	-	1.27 ± 0.06	2.29 ± 0.09	1674 ± 141
*c*PO$_{11.2}$	-	2.48 ± 0.15	12.35 ± 1.06	3034 ± 91
*r*OBC$_{7.7}$	-	4.32 ± 0.12	24.8 ± 1.7	1950 ± 83
*c*OBC$_{10.4}$	-	4.21 ± 0.16	16.86 ± 0.8	2769 ± 110

[a] Strength at Yield

Supplementary Table 2. Young's modulus of selected samples.

Sample	*r*PO$_0$-1	*r*PO$_{3.1}$	cHDPE	cLLDPE
Young's modulus (MPa)	742.7±73.8	214.2±40.5	847.3±59.7	215.4±13.4

Supplementary Table 3. Data of lap shear analysis

Polymer	Sheets	Displacement (*mm*)	Ultimate lap shear force (*N*)
*r*PO$_0$-1	PEEK	0.36 ± 0.03	244 ± 6.35
*r*PO$_{3.1}$	PEEK	0.28 ± 0.03	208 ± 13.3
*r*PO$_{9.6}$	PET	0.81 ± 0.11	207.9 ± 12.2
*c*PO$_{11.2}$	PET	0.23 ± 0.06	31.9 ± 13.8
*r*PO$_{9.6}$	PEEK	0.29 ± 0.04	201 ±18.8
*c*PO$_{11.2}$ [a]	PEEK	0.14	104.6
*r*OBC$_{7.7}$	PEEK	0.36 ± 0.02	250 ± 15.5
*c*OBC$_{10.4}$[b]	PEEK	0.07	18

[a,b] values of 1 and 2 sample testing, respectively.

References

1. Wang, G. Z., Shang, R., Cheng, W. M., Fu, Y. Irradiation-Induced Heck Reaction of Unactivated Alkyl Halides at Room Temperature. *J. Am. Chem. Soc.* **139**, 18307-18312 (2017).

2. Edwards, J. T., Merchant, R. R., McClymont, K. S., Knouse, K. W., Qin, T., Malins, L. R., Vokits, B. S., Shaw, A., Bao, D. H., Wei, F. L., Zhou, T., Eastgate, M. D., Baran, P. S. Decarboxylative Alkenylation. *Nature* **545**, 213-218 (2017).
